# Inhibition of *Toxoplasma gondii* by 1,2,4-triazole-based compounds: marked improvement in selectivity relative to the standard therapy pyrimethamine and sulfadiazine

**DOI:** 10.1080/14756366.2022.2112576

**Published:** 2022-09-27

**Authors:** Lidia Węglińska, Adrian Bekier, Nazar Trotsko, Barbara Kaproń, Tomasz Plech, Katarzyna Dzitko, Agata Paneth

**Affiliations:** a Department of Organic Chemistry, Medical University of Lublin, Lublin, Poland; b Department of Molecular Microbiology, Faculty of Biology and Environmental Protection, University of Lodz, Lodz, Poland; c Department of Clinical Genetics, Medical University of Lublin, Lublin, Poland; d Department of Pharmacology, Medical University of Lublin, Lublin, Poland

**Keywords:** *s*-triazole, anti-*Toxoplasma gondii* activity, selectivity index

## Abstract

A safer treatment for toxoplasmosis would be achieved by improving the selectivity profile of novel chemotherapeutics compared to the standard therapy pyrimethamine (PYR) and sulfadiazine (SDZ). We previously reported on the identification of the compounds with imidazole-thiosemicarbazide scaffold as potent and selective anti-*Toxoplasma gondii* (*T. gondii)* agents. In our current research, we report on the optimisation of this chemical scaffold leading to the discovery cyclic analogue **20 b** with *s*-triazole core structure. This compound displayed prominent CC_30_ to IC_50_ selectivity index (SI) of 70.72, making it 160-fold more selective than SDZ, 11-fold more selective than PYR, and 4-fold more selective than trimethoprim (TRI). Additionally, this compound possesses prerequisite drug-like anti-*Toxoplasma* properties to advance into preclinical development; it showed ability to cross the BBB, did not induce genotoxic and haemolytic changes in human cells, and as well as it was characterised by low cellular toxicity.

## 1. Introduction

Toxoplasmosis is a major parasitic disease of global importance caused by the eukaryotic pathogen *Toxoplasma gondii (T. gondii*). According to the WHO, it is estimated that up to 30% of human population is invaded by this parasite and has positive antibodies indicating toxoplasmosis^1^. It is also considered as the third most common food-borne parasitic infection requiring hospitalisation^2^. *T. gondii* is an obligate intracellular parasite endowed with a complex life cycle during which the parasite has the ability to differentiate from tachyzoites (rapidly replicating form) to the bradyzoites (slowly multiplying latent form) that are enclosed in a tissue cysts, and *vice versa*. The main routes of infection in humans include: (a) consumption of oocysts with contaminated food, water, vegetables, fruits, etc.; (b) ingestion of tissue cysts with raw or undercooked meat; (c) congenital transmission from infected mother to foetus; (d) blood transfusion or organ transplant from an infected donor^3^. In immunocompetent patients toxoplasmosis rarely requires drug treatment; the developed humoral and cellular response quickly reduce the intense proliferation of tachyzoites, therefore in most cases *Toxoplasma* infection is usually asymptomatic^4^. However, when the parasite is not eliminated from the host, it results in the long-lasting presence of tissue cysts, located mainly in the central nervous system (CNS), muscles, and the eye^1^. The long-term presence of the parasite carries the risk of permanent damage to the eye, the brain, and is also correlated with the occurrence of serious nervous disorders; schizophrenia, Parkinson’s disease, or epilepsy^5–10^. Toxoplasmosis is also associated with serious consequences in people with immune system dysfunction (HIV-positive people, AIDS patients, chemotherapy/transplant patients)^11–15^. It is included in the list of HIV-associated diseases, as can be exacerbated in the later stages of HIV infection, causing mostly severe lesions in the form of encephalitis^16–18^. In pregnant women in turn, it can cause foetal defects and even miscarriages^19–21^. Newborns with congenital toxoplasmosis are characterised mainly by pathological changes within the CNS^22–24^. In congenitally infected patients, the effects of *Toxoplasma* infection are often noticeable only several years later and these include the ocular and CNS abnormalities, endocrine disorders, or abnormal sexual development^25^.

Current first-line treatment for acute toxoplasmosis, approved by the FDA in the 1950s^26^, remains a combination of the antifolate pyrimethamine (PYR) and antibiotic sulfadiazine (SDZ), which act synergistically on the metabolic pathway of folic acid^23^^,^^27^^,^^28^, plus leucovorin to minimise host bone marrow toxicity^29^. However, due to the inhibition of the physiological folate synthesis pathway, this regimen has a number of shortcomings, including haematological side effects and embryopathies^30^. Additionally, these drugs are related to severe side effects^30–32^, drug resistance^33^, and some uncommon reactions which may be fatal^34^. In a retrospective study, 44% of patients treated with PYR, SDZ, and leucovorin required a change in the therapeutic regimen due to high rate of toxication and a number of side effects^31^. Drugs, such as macrolides, atovaquone, dapsone, and cotrimoxazole have also been used to treat clinical toxoplasmosis. However, they are poorly tolerated and have no effect on the bradyzoite form of the parasite^35–38^. Therefore, there is a longstanding demand for the development of a new generation of anti-*Toxoplasma* drugs that address these deficits.

Due to the long-term treatment of toxoplasmosis, the compounds of interest for the development of novel anti-*Toxoplasma* drugs include those with limited side effects and selective pressure on the parasite. We have previously reported^39^^,^^40^ that the imidazole-thiosemicarbazide scaffold appears promising for anti-*Toxoplasma* drug development. Several compounds more potent and more selective *in vitro* than SDZ and trimethoprim (TRI) were identified. Significant issues, however, with their solubility and toxicity have been observed in some cases. In an effort to discover more potent analogues, we have designed, synthesised, and assessed for anti-*T. gondii* activities a set of their cyclic derivatives with 1,2,4-triazole core structure. Within this chemical series we have identified a preclinical lead candidate **20b** with prominent selective inhibition of *T. gondii* parasite (SI = 70.72). Additionally, this compound possesses ability to cross the blood–brain barrier (BBB), along with prerequisite drug-like anti-*Toxoplasma* properties to advance into preclinical development; it is characterised by low cellular toxicity, lack of haemolytic potency, and genotoxic effects in human cells. In this article details of these preclinical data are reported.

## 2. Materials and methods

### 2.1. Chemistry

**Figure 1.**
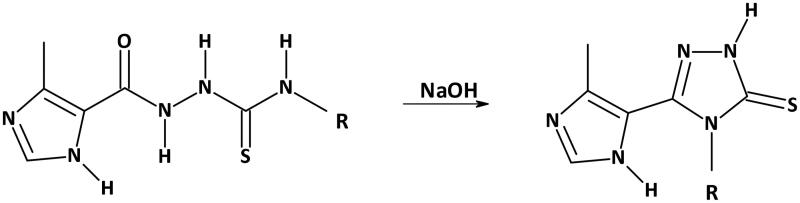
Synthetic route for 4-substituted-5–(4-methylimidazol-5-yl)-1,2,4-triazole-3-thiones **1b**–**27b**. R = *o*-FPh (**1**), *m*-FPh (**2**), *p*-FPh (**3**), *o*-ClPh (**4**), *m*-ClPh (**5**), *p*-ClPh (**6**), *o*-BrPh (**7**), *m*-BrPh (**8**), *p*-BrPh (**9**), *o*-IPh (**10**), *m*-IPh (**11**), *p*-IPh (**12**), 2,5-diFPh (**13**), *o*-MePh (**14**), *m*-MePh (**15**), *p*-MePh (**16**), 3-Cl-4-MePh (**17**), *o*-MeOPh (**18**), *m*-MeOPh (**19**), *p*-MeOPh (**20**), 3,4-diMeOPh (**21**), 4-EtOPh (**22**), *m*-AcPh (**23**), 2,6-di-*iso*-PrPh (**24**), *p*-diMeaminoPh (**25**), *p*-diEtaminoPh (**26**), Bz (**27**).

Synthesis of the *s*-triazoles (**1b**–**27b**) was conducted according to the routine one-step synthetic route^41^ shown in Figure 1. The appropriate substrates (i.e. thiosemicarbazides **1a**–**27a**) were prepared according to the procedures described in details, respectively^39^^,^^40^. All chemicals and reagents were purchased from Sigma-Aldrich (Saint Louis, MO) and Alfa Aesar (Karlsruhe, Germany) and were used as received. Melting points were determined on a Fischer-Johns block (Fisher Scientific, Schwerte, Germany) and are uncorrected. Nuclear magnetic resonance (NMR) spectroscopy was recorded on a Bruker 300 spectrometer and chemical shifts are expressed as δ (ppm). Elemental analyses were determined by a AMZ-CHX elemental analyser (PG, Gdańsk, Poland). Physicochemical characterisation of **1b**, **3b**, **16b**, and **20b** was described previously^41^^,^^42^.

#### 2.1.1. General procedure for synthesis of the 4-substituted-5–(4-methylimidazol-5-yl)-1,2,4-triazole-3-thiones 1b–27b

A solution of appropriate thiosemicarbazide (0.01 mol) in 2% NaOH was refluxed for 2 h. After cooling, the reaction mixture was filtered and the isolated solution was acidified with 3 M HCl whereupon a solid separated out. The precipitate was filtered, dried, and crystallised from ethanol.

*2.1.1.1. 4–(3-fluorophenyl)-5–(4-methylimidazol-5-yl)-1,2,4-triazole-3-thione* ***2b***.

Yield: 85% (white solid); mp 284.0 °C. ^1^H NMR (300 MHz, DMSO-d_6_): δ 2.32 (s, 3H, CH_3_), 7.08–7.12 (m, 1H, 1 × CH_ar_), 7.19–7.30 (m, 2H, 2 × CH_ar_), 7.42 (s, 1H, 1 × CH_ar_), 7.44–7.49 (m, 1H, 1 × CH_ar_), 12.35, 13.95 (2 s, 2H, 2 × NH). ^13 ^C NMR (151 MHz, DMSO-d_6_): δ 10.72, 116.08, 116.70, 125.56, 130.45, 135.12, 137.20, 137.27, 147.38, 161.20, 162.82, 168.12. Anal. Calcd for C_12_H_10_FN_5_S: C 52.35, H 3.66, N 25.44. Found: C 52.07, H 3.92, N 25.31.

*2.1.1.2. 4–(2-chlorophenyl)-5–(4-methylimidazol-5-yl)-1,2,4-triazole-3-thione* ***4b***

Yield: 81% (white solid); mp 243.4 °C. ^1^H NMR (300 MHz, DMSO-d_6_): δ 2.39 (s, 3H, CH_3_), 7.38 (s, 1H, 1 × CH_ar_), 7.40–7.49 (m, 3H, 3 × CH_ar_), 7.56–7.58 (m, 1H, 1 × CH_ar_), 12.36, 13.94 (2 s, 2H, 2 × NH). ^13 ^C NMR (75 MHz, DMSO-d_6_): δ 10.94, 123.39, 128.08, 129.55, 130.00, 131.15, 131.82, 132.72, 133.98, 135.08, 147.48, 168.07. Anal. Calcd for C_12_H_10_ClN_5_S: C 49.40, H 3.45, N 24.00. Found: C 49.25, H 3.14, N 24.28.

*2.1.1.3. 4–(3-chlorophenyl)-5–(4-methylimidazol-5-yl)-1,2,4-triazole-3-thione* ***5b***

Yield: 82% (white solid); mp 297.8 °C. ^1^H NMR (300 MHz, DMSO-d_6_): δ 2.34 (s, 3H, CH_3_), 7.23–7.25 (m, 1H, 1 × CH_ar_), 7.42–7.47 (m, 3H, 3 × CH_ar_), 7.48–7.50 (m, 1H, 1 × CH_ar_), 12.36, 13.96 (2 s, 2H, 2 × NH). ^13 ^C NMR (75 MHz, DMSO-d_6_): δ 10.77, 128.17, 129.24, 129.30, 130.54, 130.58, 132.92, 135.09, 135.16, 137.18, 147.32, 168.11. Anal. Calcd for C_12_H_10_ClN_5_S: C 49.40, H 3.45, N 24.00. Found: C 49.14, H 3.38, N 23.85.

*2.1.1.4. 4–(4-chlorophenyl)-5–(4-methylimidazol-5-yl)-1,2,4-triazole-3-thione* ***6b***

Yield: 95% (white solid); mp 255.8 °C. ^1^H NMR (300 MHz, DMSO-d_6_): δ 2.33 (s, 3H, CH_3_), 7.29–7.31 (m, 2H, 2 × CH_ar_), 7.44 (s, 1H, 1 × CH_ar_), 7.48–7.51 (m, 2H, 2 × CH_ar_), 12.38, 13.96 (2 s, 2H, 2 × NH). ^13 ^C NMR (151 MHz, DMSO-d_6_): δ 10.71, 123.10, 129.05, 129.88, 131.04, 133.64, 134.76, 135.14, 147.35, 168.13. Anal. Calcd for C_12_H_10_ClN_5_S: C 49.40, H 3.45, N 24.00. Found: C 49.29, H 3.09, N 24.16.

*2.1.1.5. 4–(2-bromophenyl)-5–(4-methylimidazol-5-yl)-1,2,4-triazole-3-thione* ***7b***

Yield: 74% (white solid); mp 235.6 °C. ^1^H NMR (300 MHz, DMSO-d_6_): δ 2.39 (s, 3H, CH_3_), 7.37–7.42 (m, 3H, 3 × CH_ar_), 7.46–7.49 (m, 1H, 1 × CH_ar_), 7.70–7.71 (dd, 1H, 1 × CH_ar_), 12.32, 13.91 (2 s, 2H, 2 × NH). ^13 ^C NMR (75 MHz, DMSO-d_6_): δ 10.71, 123.10, 129.05, 129.88, 131.04, 133.64, 134.76, 135.14, 147.35, 168.13. Anal. Calcd for C_12_H_10_BrN_5_S: C 42.87, H 3.00, N 20.83. Found: C 42.98, H 3.31, N 20.53.

*2.1.1.6. 4–(3-bromophenyl)-5–(4-methylimidazol-5-yl)-1,2,4-triazole-3-thione* ***8b***

Yield: 72% (white solid); mp 299.7 °C. ^1^H NMR (300 MHz, DMSO-d_6_): δ 2.33 (s, 3H, CH_3_), 7.25–7.29 (m, 1H, 1 × CH_ar_), 7.36–7.41 (m, 1H, 1 × CH_ar_), 7.44 (s, 1H, 1 × CH_ar_), 7.53–7.54 (m, 1H, 1 × CH_ar_), 7.60–7.63 (m, 1H, 1 × CH_ar_), 12.35, 13.96 (2 s, 2H, 2 × NH). ^13 ^C NMR (75 MHz, DMSO-d_6_): δ10.75, 121.12, 123.05, 128.54, 129.92, 130.83, 131.98, 132.09, 135.13, 137.24, 147.26, 168.08. Anal. Calcd for C_12_H_10_BrN_5_S: C 42.87, H 3.00, N 20.83. Found: C 43.22, H 2.88, N 20.97.

*2.1.1.7. 4–(4-bromophenyl)-5–(4-methylimidazol-5-yl)-1,2,4-triazole-3-thione* ***9b***

Yield: 91% (white solid); mp 265.9 °C. ^1^H NMR (300 MHz, DMSO-d_6_): δ 2.34 (s, 3H, CH_3_), 7.22–7.24 (m, 2H, 2 × CH_ar_), 7.43 (s, 1H, 1 × CH_ar_), 7.61–7.64 (m, 2H, 2 × CH_ar_), 12.40, 13.96 (2 s, 2H, 2 × NH). ^13 ^C NMR (75 MHz, DMSO-d_6_): δ 10.71, 122.25, 123.08, 129.89, 131.34, 132.00, 135.12, 135.20, 147.29, 168.04. Anal. Calcd for C_12_H_10_BrN_5_S: C 42.87, H 3.00, N 20.83. Found: C 43.15, H 3.35, N 20.56.

*2.1.1.8. 4–(2-iodophenyl)-5–(4-methylimidazol-5-yl)-1,2,4-triazole-3-thione* ***10b***

Yield: 92% (white solid); mp 297.6 °C. ^1^H NMR (300 MHz, DMSO-d_6_): δ 2.39 (s, 3H, CH_3_), 7.17–7.20 (m, 1H, 1 × CH_ar_), 7.37–7.39 (m, 1H, 1 × CH_ar_), 7.41 (s, 1H, 1 × CH_ar_), 7.47–7.50 (m, 1H, 1 × CH_ar_), 7.87–7.89 (m, 1H, 1 × CH_ar_), 12.45, 13.90 (2 s, 2H, 2 × NH). ^13 ^C NMR (75 MHz, DMSO-d_6_): δ 11.01, 100.71, 123.29, 129.23, 129.57, 131.07, 131.28, 135.01, 138.80, 139.22, 147.01, 167.81. Anal. Calcd for C_12_H_10_IN_5_S: C 37.61, H 2.63, N 18.28. Found: C 37.29, H 2.89, N 18.09.

*2.1.1.9. 4–(3-iodophenyl)-5–(4-methylimidazol-5-yl)-1,2,4-triazole-3-thione* ***11b***

Yield: 86% (white solid); mp 276.5 °C. ^1^H NMR (300 MHz, DMSO-d_6_): δ 2.34 (s, 3H, CH_3_), 7.21–7.24 (m, 1H, 1 × CH_ar_), 7.27–7.29 (m, 1H, 1 × CH_ar_), 7.44 (s, 1H, 1 × CH_ar_), 7.66 (m, 1H, 1 × CH_ar_), 7.77–7.79 (m, 1H, 1 × CH_ar_), 12.34, 13.94 (2 s, 2H, 2 × NH). ^13 ^C NMR (75 MHz, DMSO-d_6_): δ 10.76, 93.96, 128.85, 130.86, 135.10, 136.97, 137.43, 137.48, 137.80, 137.84, 147.27, 168.06. Anal. Calcd for C_12_H_10_IN_5_S: C 37.61, H 2.63, N 18.28. Found: C 37.37, H 2.92, N 18.56.

*2.1.1.10. 4–(4-iodophenyl)-5–(4-methylimidazol-5-yl)-1,2,4-triazole-3-thione* ***12b***

Yield: 89% (white solid); mp 290.0 °C. ^1^H NMR (300 MHz, DMSO-d_6_): δ 2.33 (s, 3H, CH_3_), 7.06–7.08 (m, 2H, 2 × CH_ar_),7.44 (s, 1H, 1 × CH_ar_), 7.77–7.80 (m, 2H, 2 × CH_ar_), 12.39, 13.95 (2 s, 2H, 2 × NH). ^13 ^C NMR (75 MHz, DMSO-d_6_): δ 10.71, 95.59, 123.08, 129.88, 131.34, 135.16, 135.65, 137.86, 147.23, 168.04. Anal. Calcd for C_12_H_10_IN_5_S: C 37.61, H 2.63, N 18.28. Found: C 37.34, H 2.78, N 18.18.

*2.1.1.11. 4–(2,5-difluorophenyl)-5–(4-methylimidazol-5-yl)-1,2,4-triazole-3-thione* ***13b***.

Yield: 87% (white solid); mp 253.9 °C. ^1^H NMR (300 MHz, DMSO-d_6_): δ 2.41 (s, 3H, CH_3_), 7.37–7.44 (m, 4H, 4 × CH_ar_), 12.45, 14.02 (2 s, 2H, 2 × NH). ^13 ^C NMR (75 MHz, DMSO-d_6_): δ 10.93, 117.36 (d, *J* = 8.9 Hz), 118.49 (d, *J* = 27.1 Hz), 123.22, 125.05, 129.75, 135.19, 147.45, 153.24, 156.26, 159.44, 168.21. Anal. Calcd for C_12_H_9_F_2_N_5_S: C 49.14, H 3.09, N 23.88. Found: C 48.97, H 3.19, N 23.57.

*2.1.1.12. 5–(4-methylimidazol-5-yl)-4–(2-methylphenyl)-1,2,4-triazole-3-thione* ***14b***

Yield: 92% (white solid); mp 249.9 °C. ^1^H NMR (300 MHz, DMSO-d_6_): δ 2.04 (s, 3H, CH_3_), 2.34 (s, 3H, CH_3_), 7.06–7.08 (m, 1H, 1 × CH_ar_), 7.20–7.24 (m, 1H, 1 × CH_ar_), 7.29–7.33 (m, 2H, 2 × CH_ar_), 7.38 (s, 1H, 1 × CH_ar_), 12.31, 13.90 (2 s, 2H, 2 × NH). ^13 ^C NMR (75 MHz, DMSO-d_6_): δ 10.70, 18.05, 123.39, 126.81, 129.28, 129.51, 130.74, 135.17, 135.22, 135.44, 136.78, 147.60, 167.76. Anal. Calcd for C_13_H_13_N_5_S: C 57.54, H 4.83, N 25.81. Found: C 57.19, H 4.63, N 25.99.

*2.1.1.13. 5–(4-methylimidazol-5-yl)-4–(3-methylphenyl)-1,2,4-triazole-3-thione* ***15b***

Yield: 86% (white solid); mp 248.7 °C. ^1^H NMR (300 MHz, DMSO-d_6_): δ 2.28 (s, 3H, CH_3_), 2.30 (s, 3H, CH_3_), 7.00–7.02 (m, 1H, 1 × CH_ar_), 7.07–7.08 (m, 1H, 1 × CH_ar_), 7.20–7.22 (m, 1H, 1 × CH_ar_), 7.28–7.31 (t, 1H, 1 × CH_ar_), 7.43 (s, 1H, 1 × CH_ar_), 12.30, 13.91 (2 s, 2H, 2 × NH). ^13 ^C NMR (75 MHz, DMSO-d_6_): δ 10.64, 21.25, 123.08, 126.06, 128.78, 129.33, 129.75, 135.09, 135.12, 135.56, 138.27, 147.48, 168.19. Anal. Calcd for C_13_H_13_N_5_S: C 57.54, H 4.83, N 25.81. Found: C 57.24, H 4.67, N 25.65.

*2.1.1.14. 4–(3-chloro-4-methylphenyl)-5–(4-methylimidazol-5-yl)-1,2,4-triazole-3-thione* ***17b***

Yield: 92% (white solid); mp 264.1 °C. ^1^H NMR (300 MHz, DMSO-d_6_): δ 2.34 (s, 3H, CH_3_), 2.37 (s, 3H, CH_3_), 7.12–7.14 (dd, 1H, 1 × CH_ar_), 7.38–7.40 (m, 2H, 2 × CH_ar_), 7.44 (s, 1H, 1 × CH_ar_), 12.37, 13.94 (2 s, 2H, 2 × NH). ^13 ^C NMR (75 MHz, DMSO-d_6_): δ 10.74, 19.84, 123.17, 127.98, 129.39, 129.87, 131.39, 132.97, 134.72, 135.17, 136.48, 147.35, 168.15. Anal. Calcd for C_13_H_12_ClN_5_S: C 51.06, H 3.96, N 22.90. Found: C 51.36, H 4.24, N 23.09.

*2.1.1.15. 4–(2-methoxyphenyl)-5–(4-methylimidazol-5-yl)-1,2,4-triazole-3-thione* ***18b***

Yield: 94% (white solid); mp 145.2 °C. ^1^H NMR (300 MHz, DMSO-d_6_): δ 2.31(s, 3H, CH_3_), 3.58 (s, 3H, OCH_3_), 6.98–7.00 (td, 1H, 1 × CH_ar_), 7.07–7.09 (dd, 1H, 1 × CH_ar_), 7.19–7.21 (dd, 1H, 1 × CH_ar_), 7.37–7.40 (m, 2H, 2 × CH_ar_), 12.33, 13.80 (2 s, 2H, 2 × NH). ^13 ^C NMR (75 MHz, DMSO-d_6_): δ 10.78, 56.11, 112.85, 120.55, 123.45, 124.68, 129.24, 130.59, 130.81, 134.87, 148.03, 155.43, 168.37. Anal. Calcd for C_13_H_13_N_5_OS: C 54.34, H 4.56, N 24.37. Found: C 54.03, H 4.56, N 24.03.

*2.1.1.16. 4–(3-methoxyphenyl)-5–(4-methylimidazol-5-yl)-1,2,4-triazole-3-thione* ***19b***

Yield: 90% (white solid); mp 228.8 °C. ^1^H NMR (300 MHz, DMSO-d_6_): δ 2.28 (s, 3H, CH_3_), 3.37 (s, 3H, OCH_3_), 6.79–6.82 (m, 1H, 1 × CH_ar_), 6.84–6.85 (m, 1H, 1 × CH_ar_), 6.97–6.99 (m, 1H, 1 × CH_ar_), 7.30–7.33 (m, 1H, 1 × CH_ar_), 7.44 (s, 1H, 1 × CH_ar_), 12.32, 13.92 (2 s, 2H, 2 × NH). ^13 ^C NMR (75 MHz, DMSO-d_6_): δ 10.62, 55.75, 114.42, 115.18, 121.17, 123.09, 129.70, 135.15, 136.60, 136.82, 147.46, 159.51, 168.13. Anal. Calcd for C_13_H_13_N_5_OS: C 54.34, H 4.56, N 24.37. Found: C 54.52, H 4.30, N 24.04.

*2.1.1.17. 4–(3,4-dimethoxyphenyl)-5–(4-methylimidazol-5-yl)-1,2,4-triazole-3-thione* ***21b***

Yield: 89% (grey solid); mp 213.4 °C. ^1^H NMR (300 MHz, DMSO-d_6_): δ 2.26 (s, 3H, CH_3_), 3.67, 3.78 (2 s, 6H, 2 × OCH_3_), 6.76–6.78 (dd, 1H, 1 × CH_ar_), 6.87(d, 1H, 1 × CH_ar_), 6.95–6.96 (d, 1H, 1 × CH_ar_), 7.46 (s, 1H, 1 × CH_ar_), 12.28, 13.88 (2 s, 2H, 2 × NH). ^13 ^C NMR (75 MHz, DMSO-d_6_): δ 10.63, 55.93, 111.27, 113.00, 121.20, 123.29, 128.17, 129.72, 135.15, 147.69, 148.55, 149.14, 168.33. Anal. Calcd for C_14_H_15_N_5_O_2_S: C 52.98, H 4.76, N 22.07. Found: C 53.27, H 4.54, N 22.36.

*2.1.1.18. 4–(4-ethoxyphenyl)-5–(4-methylimidazol-5-yl)-1,2,4-triazole-3-thione* ***22b***

Yield: 92% (grey solid); mp 239.9 °C. ^1^H NMR (300 MHz, DMSO-_d6_): δ 1.31–1.35(t, 3H, CH_3_), 2.25 (s, 3H, CH_3_), 4.00–4.06 (q, 2H, 1 × CH_2_), 6.90–6.94 (m, 2H, 2 × CH_ar_), 7.10–7.15 (m, 2H, 2 × CH_ar_), 7.43 (s, 1H, 1 × CH_ar_), 12.38, 13.87 (2 s, 2H, 2 × NH). ^13 ^C NMR (151 MHz, DMSO-d_6_): δ 10.60, 15.11, 63.71, 114.48, 123.05, 128.09, 130.11, 135.10, 135.14, 147.66, 158.80, 168.41. Anal. Calcd for C_14_H_15_N_5_OS: C 55.80, H 5.02, N 23.24. Found: C 55.44, H 4.87, N 23.02.

*2.1.1.19. 4–(3-acetylphenyl)-5–(4-methylimidazol-5-yl)-1,2,4-triazole-3-thione* ***23b***

Yield: 94% (white solid); mp 151.9 °C. ^1^H NMR (300 MHz, DMSO-d_6_): δ 2.34 (s, 3H, CH_3_), 2.57 (s, 3H, COCH_3_), 7.40 (s, 1H, 1 × CH_ar_), 7.53–7.55 (m, 1H, 1 × CH_ar_), 7.58–7.60 (t, 1H, 1 × CH_ar_), 7.85 (t, 1H, 1 × CH_ar_), 8.00–8.02 (dt, 1H, 1 × CH_ar_), 12.41, 14.00 (2 s, 2H, 2 × NH). ^13 ^C NMR (75 MHz, DMSO-d_6_): δ 10.74, 27.27, 123.12, 128.70, 129.06, 129.47, 129.90, 133.91, 135.12, 136.25, 137.47, 147.35, 168.10, 197.65. Anal. Calcd for C_14_H_13_N_5_OS: C 56.17, H 4.38, N 23.40. Found: C 55.89, H 4.17, N 23.13.

*2.1.1.20. 4–(2,6-diisopropylphenyl)-5–(4-methylimidazol-5-yl)-1,2,4-triazole-3-thione* ***24b***

Yield: 74% (white solid); mp 251.9 °C. ^1^H NMR (300 MHz, DMSO-d_6_): δ 0.88–0.89 (d, 6H, 2 × CH_3(isoPr)_), 1.15–1.16 (d, 6H, 2 × CH_3(isoPr)_), 2.34–2.39 (m, 5H, 2 × CH, 1 × CH_3_), 7.18–7.19 (d, 2H, 2 × CH_ar_), 7.35–7.38 (m, 2H, 2 × CH_ar_), 12.37, 13.87 (2 s, 2H, 2 × NH). ^13 ^C NMR (75 MHz, DMSO-d_6_): δ 10.76, 23.27, 28.95, 123.75, 123.81, 129.41, 129.92, 131.47, 134.83, 146.29, 148.36, 168.71. Anal. Calcd for C_18_H_23_N_5_S: C 63.31, H 6.79, N 20.51. Found: C 63.62, H 6.66, N 20.34.

*2.1.1.21. 5–(4-methylimidazol-5-yl)-4–(4-dimethyloaminophenyl)-1,2,4-triazole-3-thione* ***25b***

Yield: 92% (grey solid); mp 288.2 °C. ^1^H NMR (300 MHz, DMSO-d_6_): δ 2.23 (s, 3H, CH_3_), 2.93 (s, 6H, 2 × CH_3_), 6.66–6.68 (m, 2H, 2 × CH_ar_), 6.99–7.02(m, 2H, 2 × CH_ar_), 7.45 (s, 1H, 1 × CH_ar_), 12.28, 13.81 (2 s, 2H, 2 × NH). ^13 ^C NMR (75 MHz, DMSO-d_6_): δ 10.52, 40.43, 111.84, 122.85, 123.88, 129.22, 135.12, 135.22, 147.97, 150.35, 168.54. Anal. Calcd for C_14_H_16_N_6_S: C 55.98, H 5.37, N 27.98. Found: C 56.36, H 5.33, N 28.06.

*2.1.1.22. 4–(4-diethylaminophenyl)-5–(4-methylimidazol-5-yl)-1,2,4-triazole-3-thione* ***26b***

Yield: 94% (white solid); mp 147.0 °C. ^1^H NMR (300 MHz, DMSO-d_6_): δ 1.09–1.11(t, 6H, 2 × CH_3_), 2.23 (s, 3H, CH_3_), 3.32–3.34(q, 4H, 2 × CH_2_), 6.59–6.63 (m, 2H, 2 × CH_ar_), 6.96–6.98 (m, 2H, 2 × CH_ar_), 7.46 (s, 1H, 1× CH_ar_), 12.34, 13.79 (2 s, 2H, 2 × NH). ^13 ^C NMR (151 MHz, DMSO-d_6_): δ 10.42, 12.90, 40.08, 110.75, 122.82, 123.77, 129.14, 129.51, 135.10, 147.64, 148.08, 168.58 Anal. Calcd for C_16_H_20_N_6_S: C 58.51, H 6.14, N 25.59. Found: C 58.42, H 5.78, N 25.81.

*2.1.1.23. 4-benzyl-5–(4-methylimidazol-5-yl)-1,2,4-triazole-3-thione* ***27b***

Yield: 84% (white solid); mp 229.5 °C. ^1^H NMR (300 MHz, DMSO-d_6_): δ 2.34 (s, 3H, CH_3_), 5.76 (s, 2H, CH_2_), 7.15–7.26 (m, 5H, 5 × CH_ar_), 7.70 (s, 1H, 1 × CH_ar_), 12.49, 13.83 (2 s, 2H, 2 × NH). ^13 ^C NMR (151 MHz, DMSO-d_6_): δ 10.94, 47.06, 123.93, 127.63, 128.61, 129.52, 135.22, 135.27, 137.21, 147.32, 167.31. Anal. Calcd for C_13_H_13_N_5_S: C 57.54, H 4.83, N 25.81. Found: C 57.23, H 4.78, N 25.96.

### 2.2. Compounds and drugs preparation

All experiments were carried out in BSL-2 laboratory with biological safety cabinet. Compounds **1b**–**27b** were dissolved in dimethyl sulfoxide (DMSO, Sigma-Aldrich, St. Louis, MO) to 125 mM. TRI and PYR (92131 and PYR, 46706, Sigma-Aldrich) were dissolved in DMSO to 25 mM. The final concentration of DMSO in compounds and drugs dilution was below 1.00%. SDZ sodium salt (SDZ, S6387, Sigma-Aldrich) was dissolved in DPBS (Dulbecco’s Phosphate Buffered Saline [PBS], D8537, Sigma) without calcium chloride and magnesium chloride. All dilutions were freshly prepared before experiments.

### 2.3. Cell and parasite culture

The L929 mouse fibroblast (ATTC® CCL-1^™^, Manassas, VA) was routinely maintained in RPMI 1640 media (R8758, Sigma-Aldrich), supplemented with 10% foetal bovine serum (FBS, ATCC^®^ 30–2020^™^), 100 I.U./mL penicillin and 100 μg/mL streptomycin (Penicillin-Streptomycin Solution ATCC^®^ 30–2300^™^). Cells were trypsinised (Trypsin-EDTA Solution, 1X ATCC^®^ 30–2101^™^) twice a week and seeded at a density of 1 × 10^6^ per T25 cell culture flask and incubated at 37 °C and 10% CO_2_ to achieve a confluent monolayer. The Hs27 (human foreskin fibroblast) (ATCC^®^ CRL-1634^™^) were culturing in DMEM (Dulbecco’s Modified Eagle’s Medium-ATCC^®^ 30–2002^™^) supplemented with 10% FBS, 100 I.U./mL penicillin and 100 μg/mL streptomycin. Cells were trypsinised twice a week and seeded at a density of 1 × 10^6^ per T25 cell culture flask and incubated in a 37 °C and 5% CO_2_ to achieve a confluent monolayer. The RH strain of *T. gondii* (RH-GFP ATCC^®^ 50940^™^, highly virulent, haplogroup first, with expression of green fluorescent protein) was maintained as tachyzoites, in parasite culture medium, which contains DMEM medium with 3% HIFBS (Heat-Inactivated FBS; 1 h in 56 °C). Infected tissue culture cells were incubated in a 37 °C and 5% CO_2_.

### 2.4. Cell viability assay

Cell viability assay was performed according to international standards (ISO 10993–5:2009(E)), using tetrazolium salt (MTT, Sigma-Aldrich) and mouse fibroblasts L929 cells. Culture medium RPMI 1640, without phenol red (Biowest, Nuaille, France) supplemented with 10% FBS, 2 mM L-glutamine (Sigma-Aldrich), 100 I.U./mL penicillin, 100 μg/mL streptomycin were used. Briefly, 1 × 10^4^/well of L929 cells were placed in 96-well plates and incubated for 24 h at 37 °C and 10% CO_2_. Afterwards, the old culture medium was replaced with 100 μL of the compound, drug or inhibitor, diluted in culture medium and the cells treated for 24 h. Additionally, cells were treated with 0.03–4.0% concentration of DMSO as the solvent (data not shown). Then, 50 μL of 1 mg/mL of MTT solution in RPMI 1640 without phenol red was added to each well and incubated for 2 h at 37 °C and 10% CO_2_. Next, cell culture medium was aspirated carefully and 150 μL of DMSO was added to each well, and the plates were gently mixed. Then, 25 μL 0.1 M glycine buffer (pH 10.5) (Sigma-Aldrich) was added. The optical density at 570 nm using a multi-mode microplate reader SpectraMax^®^ i3 (Syngen, Taipei City, Taiwan) was recorded. The results were expressed as a percentage of viability compared to untreated cells. All experiments were performed in triplicate.

### 2.5. Antiparasitic assay

The influence of **1b**–**27b** and drugs on *T. gondii* RH-GFP proliferation was performed as follows: 1 × 10^4^ per well of Hs27 cells were seeded on black, 96-well, tissue culture-treated plates with optical bottoms (Corning, NY) in DMEM cell culture medium. After 72 h of incubation, the medium was removed and then 1 × 10^5^ per well of tachyzoites of the RH strain were added to the cell monolayers, in DMEM without phenol red with 3% HIFBS. One hour later, the compounds and drugs dilutions in the DMEM without phenol red medium were added to the Hs27 cells with *T. gondii*. Concentration of the compounds was below cytotoxic concentration (<CC_30_). After a subsequent 96 h of incubation, the plates were read, and both excitation (475 nm) and emission (509 nm) from the bottom using multi-mode microplate reader SpectraMax^®^ i3. The results were expressed as mean fluorescent intensity (MFI) and transformed to the percentage of viability compared to untreated cells. Finally, the inhibitory concentrations for 50% inhibition of *T. gondii* proliferation (IC_50_*_Tg_*) were calculated. All experiments were performed in triplicate.

### 2.6. Influence of compounds during toxoplasma growth (Assay A and B)

The Hs27 cells were cultured on Lab-Tek^™^ 4-well Chamber Slides (Nunc) (5 × 10^4^ cells/500 μL per well).

Assay A – after 48 h, the medium was removed and then 5 × 10^5^/500 µL/well tachyzoites of the RH-GFP strain were added to the cell monolayers, in parasite culture medium with **20b** at 1 × IC_50_*_Tg_*, 2 × IC_50_*_Tg_*, and 5 × IC_50_*_Tg_* concentration, for 3 h. Then, the cells were washed to remove extracellular parasites and parasite culture medium (without any compounds) was added for 24 h.

Assay B – after 48 h, the medium was removed and then 5 × 10^5^/500 µL/well tachyzoites of the RH-GFP strain were added to the cell monolayers, in parasite culture medium for 3 h. Then, the cells were washed to remove extracellular parasites, and the cells were treated with **20b** at 1 × IC_50_*_Tg_*, 2 × IC_50_*_Tg_*, and 5 × IC_50_*_Tg_* concentration, for 24 h.

In both assays as the control, Hs27 cells were infected, but not treated. After subsequent 24 h of incubation (Assay A and B), the slides were washed with sterile PBS, fixed with formaldehyde solution (252549, Sigma-Aldrich), 3.7% in PBS for 20 min, and stained with DAPI for 10 min (1 mg/mL, ThermoFisher Scientific, Waltham, MA). Microscopic analysis of 100 cells in turn, comprised of counting the infected cell ratio and tachyzoites per each parasitophorous vacuoles of infected cell, was performed using a fluorescent microscope (Axio Scope.A1, Carl Zeiss, Germany) at a magnification ×1000. For image processing ZEISS ZEN Microscope Software version 3.1 (Oberkochen, Germany) was used. Three independent experiments on triplicate chamber slides using the same conditions for both assays were performed.

### 2.7. Inhibition of tyrosinase activity assay

Tyrosinase (TYR) from mushrooms (T3824, Sigma) was used in our study. We analysed inhibition of **20 b** on both monophenolase and diphenolase activity of TYR. All compound dilutions, both substrate L-tyrosine (Tyr) and levodopa (DOPA), and TYR were prepared in 50 mM phosphate buffer (pH 6.5). The volume of the reaction mixture was 200 μL and contained: 80 μL of 2.5 mM substrate (Tyr or DOPA), 50 μL of the compound dilutions (4, 40, 100, 200, 400, 800, and 1600 μM) and 70 μL of TYR (10 U). Firstly, the substrate and compound were added to a 96-well flat-bottom plate (Nunc MaxiSorp^™^, Roskilde, Denmark), next TYR was added and the initial absorbance (A0) was measured spectrophotometrically at 492 nm. Then, the plate was incubated at 25 °C for 30 min. After incubation, the amount of dopachrome produced in the reaction mixture was determined spectrophotometrically at 492 nm (A30) using the multi-mode microplate reader SpectraMax^®^ i3. As 100% TYR activity samples without addition of compound, were used. The average results from three experiments are shown.

### 2.8. Genotoxic activity evaluation

Genotoxic effect of **20b** (at the concentration representing its IC_50_*_Tg_* against RH-GFP strain) was carried out using a single cell gel electrophoresis technique (comet assay) based on OxiSelect Comet Assay Kit (Cell Biolabs, Inc., San Diego, CA). All the procedures performed were in line with the manufacturer’s protocol. Human Hs27 cells, previously exposed for 24 h to the compound, were combined with liquefied low melting Agarose at 37 °C and transferred onto the OxiSelect comet slides. After storing for 15 min at 4 °C, the slides were immersed in a pre-chilled Lysis Buffer (for 45 min at 4 °C) and Alkaline Solution (for 30 min at 4 °C). Afterwards, the Alkaline Solution was replaced with pre-chilled TBE electrophoresis solution. After immersing for 5 min, the slides were put into the horizontal electrophoresis chamber and covered with TBE electrophoresis buffer. The electrophoresis was run for 15 min at 1 volt/cm. Finally, DNA was stained with Vista Green DNA Dye for 15 min at room temperature and observed under fluorescence microscope (Olympus BX63, Tokyo, Japan). The images of cells were captured using XM10 digital camera (Olympus). The DNA damage was measured quantitatively using OpenComet software. At least 50 randomly selected images were used in each analysis.

### 2.9. Pampa-BBB assay

BBB permeability of **20b** was investigated using a parallel artificial membrane permeability assay (PAMPA) method. The PAMPA system, consisted of a 96-well microfilter plate, was divided into two chambers: a donor at the bottom and an acceptor at the top, separated by a 120-μm-thick microfilter disc coated with BBB lipid solution (Pion, Inc., Billerica, MA). The solutions of **20b** were prepared in dimethylsulphoxide (DMSO) at 4 mg/mL concentration and then diluted with Prisma buffer (pH = 7.4) to obtain the donor drug solution with the final nominal concentration of 20 μg/mL. Then, 180 μL of the donor solution were added to the donor wells. Subsequently, each filter membrane of the top plate was coated with 5 μL BBB-1 lipid solution (Pion Inc.) and the acceptor well was filled with 200 μL of Brain Sink Buffer (BSB). The acceptor plate and the donor plate were sandwiched together and incubated at 37 °C for 180 min.

The permeability coefficient value (P_e_) was calculated by using the following equation:

$$P_{e}=\frac{-\ln\left(1-\frac{C_{A}}{C_{\text{equilibrium}}}\right)}{S\times \left(\frac{1}{V_{D}}+\frac{1}{V_{A}}\right)\times t}$$

where *V_D_* – donor volume, *V_A_* – acceptor volume, *C*_equilibrium_ – equilibrium concentration, $C_{\text{equilibrium}}=\frac{C_{D}\times V_{D}+C_{A}\times V_{A}}{V_{D}+V_{A}},$ *C_D_* – donor concentration, *C_A_* – acceptor concentration, *S* – membrane area, and *t* – incubation time (in seconds).

### 2.10. Haemolytic activity determination

Human red blood cells (RBCs) concentrate was obtained from the Regional Blood Donation and Transfusion Centre (Lublin, Poland). RBCs concentrate (5 mL) was washed three times with sterile PBS and centrifuged at 500 *g* for 3 min. The obtained pellet was resuspended using sterile PBS in order to obtain 2% suspension of RBCs, which was subsequently mixed with 1 mL of different concentrations (i.e. 5, 10, 35 µg/mL) of **20b**. The mixtures were incubated at 37 °C for 30 min and centrifuged at 1400 *g* for 10 min. The amount of free haemoglobin in supernatants was measured spectrophotometrically at 405 nm. Negative and positive controls were performed by incubating RBCs with sterile PBS and 0.1% Triton-X, respectively. Each experiment was run in triplicate.

### 2.11. Cytotoxicity of compound 20b against human glioblastoma T98G cells

Cytotoxic effect of **20b** was evaluated using glioblastoma (T98G) cells obtained from the American Type Culture Collection (Manassas, VA). Cells were cultured in Dulbecco’s Modified Eagle’s Medium (DMEM-high glucose) (Sigma Aldrich, St. Louis, MO), supplemented with 10% heat inactivated FBS, penicillin (100 U/mL), and streptomycin (100 μg/mL) (Sigma Aldrich, St. Louis, MO). Cells were maintained in a humidified atmosphere of 5% CO_2_ and 95% air at 37 °C. Stock solutions of the investigated compounds (**20b** and temozolomide) were prepared by dissolving solid substances in a sterile DMSO. T98G glioblastoma cells were seeded into 96-well sterile plates at a density of 1 × 10^5^cells/mL. After 24 h of incubation, the medium was removed from each well and then cells were incubated for the next 24 h with different concentrations of the investigated compounds (10, 25, 35, 50, and 100 µg/mL) in the medium containing 2% FBS. Viability of T98G cells was evaluated using MTT assay, whose principle is based on the conversion of 3–(4,5-dimethylthiazol-2-yl)-2,5-diphenyltetrazolium bromide (MTT) into dark-blue formazan crystals. In brief, after 24 h incubation of cells with varying concentrations of the tested compounds, culture medium was removed from the plate. Cells were washed with PBS, and then 100 µL of medium containing 10% MTT solution (5 mg/mL) was added to each well. After 3 h incubation, 100 µL (per well) of 10% SDS buffer solution was added to solubilise formazan crystals. After overnight incubation the absorbance was measured at 570 nm using a microplate reader (Epoch, BioTek Instruments, Winooski, VT). Experiments were repeated three times, and the measurements in each experiment were run in quadruplicate. Viability of the investigated cells was expressed as % of the viability of the untreated cells. DMSO in the concentrations present in the dilutions of stock solutions did not influence the viability of the tested cells.

### 2.12. In silico ADME prediction

The ADME properties of the *s*-triazoles **1b**–**27b** (i.e. water solubility, log *p*, pharmacokinetics, drug-likeness, and medicinal chemistry friendliness) were predicted using swissADME online software available at http://www.swissadme.ch.

### 2.13. Graphs and statistical analyses

Statistical analyses and graphs were performed using GraphPad Prism version 9.0.0 for macOS (GraphPad Software, San Diego, CA). For the compounds with the CC_30_ or the IC_50_ values greater than the highest concentration tested, the values were calculated based on extrapolation of the curves. Additionally, a relationship between cytotoxicity and antiparasitic activity, were calculated as the ratio of the 30% cytotoxic concentration (CC_30_) to the 50% antiparasitic concentration (IC_50_*_Tg_*) and presented as selectivity index (SI).

## 3. Results and discussion

### 3.1. In silico ADME prediction

Before we embarked on the synthesis of the targeted *s*-triazoles **1b**–**27b**, we have submitted all of them to a *in silico* SwissADME screening (http://www.swissadme.ch) to predict their water solubility, lipophilicity, pharmacokinetics, drug-likeness, and medicinal chemistry friendliness. As presented in Table S1 (see Supplemental Material), designed set of the *s*-triazoles should possess a reasonable range in key parameters influencing ADME properties, suggesting a good drug-like feature of these compounds. Thus, the targeted *s*-triazoles **1b**–**27b** were prepared according to know one-step procedure^41^ (see Materials and methods), and then submitted to the cytotoxic assay.

### 3.2. Cytotoxicity

**Figure 2.**
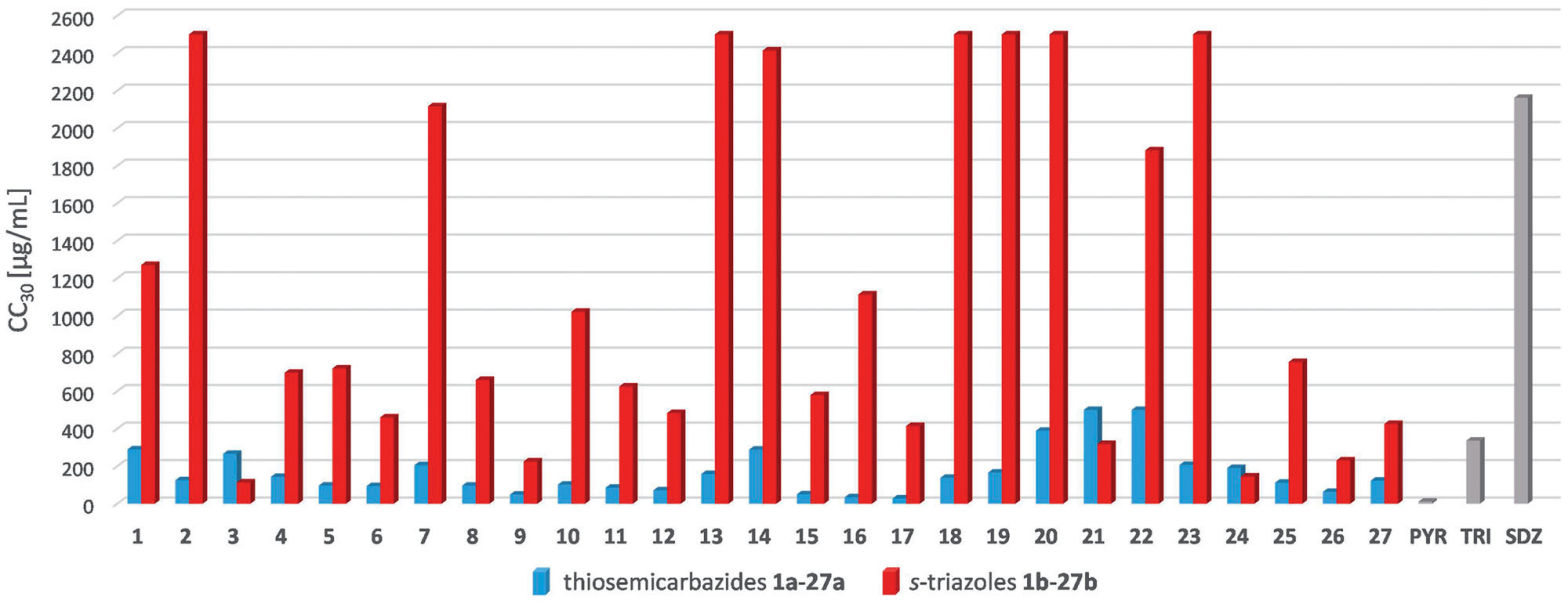
The cytotoxic effects of the thiosemicarbazides **1a**–**27a** and the *s*-triazoles **1b**–**27b** against L929 cells. The CC_30_values represent the highest dilution of the samples to cause 30% or higher destruction of the cells. These were determined based on the plotted curves using GraphPad Prism program version 9.0.0. Reference drugs: PYR: pyrimethamine; TRI: trimethoprim; SDZ: sulfadiazine. Data for **1a**–**27a** were taken from Paneth et al.^39^^,^^40^

The reported methyl thiazolyltetrazole (MTT) method was used to study *in vitro* cytotoxic profile of the *s*-triazoles **1b**–**27b** to the host cells (L929). Experiments were performed in accordance with the international standard IS0-10993–5:2009. Cytotoxicity was defined as the highest dilution of the samples to cause 30% or higher destruction of the cells (CC_30_). The CC_30_ results are graphically presented in Figure 2. Details are reported in Table 1. The data for acyclic precursors **1a**–**27a** are also included for comparison^39^^,^^40^.

**Table 1.** Cytotoxicity profile and anti-*T. gondii* activity of the thiosemicarbazides **1a**–**27a** and the *s*-triazoles **1b**–**27b**^a^.

Compd.	CC_30_^b^ [µg/mL]	IC_50_*_Tg_*^c^ [µg/mL]	SI^d^	Compd.	CC_30_ [µg/mL]	IC_50_*_Tg_*^c^ [µg/mL]	SI
**1a**	289.6	68.83	4.21	**1b**	1270.57	219.84	5.78
**2a**	125.17	113.45	1.10	**2b**	>2500.00	103.04	24.26
**3a**	266.01	110.31	2.41	**3b**	113.71	205.87	0.55
**4a**	143.15	35.61	4.02	**4b**	698.23	344.51	2.03
**5a**	96.78	25.70	4.37	**5b**	721.27	310.31	2.32
**6a**	94.12	73.37	1.28	**6b**	460.26	266.81	1.73
**7a**	205.31	27.65	7.43	**7b**	2116.90	66.74	31.72
**8a**	96.12	15.64	6.15	**8b**	660.09	103.04	6.41
**9a**	48.89	14.57	3.36	**9b**	225.58	152.83	1.48
**10a**	101.58	20.31	5.00	**10b**	1021.41	80.26	12.73
**11a**	85.57	10.3	8.31	**11b**	625.17	36.44	17.16
**12a**	71.58	22.01	3.25	**12b**	483.06	99.08	4.88
**13a**	158.49	53.7	2.95	**13b**	>2500.00	224.34	11.14
**14a**	288.4	∼150.31	1.92	**14b**	2414.35	77.32	31.23
**15a**	50.12	37.15	1.35	**15b**	579.43	79.10	7.33
**16a**	34.67	∼57.58	0.60	**16b**	1113.53	110.18	10.11
**17a**	28.18	18.62	1.51	**17b**	414.57	174.86	2.37
**18a**	138.04	33.11	4.17	**18b**	>2500.00	93.22	26.82
**19a**	165.96	79.65	2.08	**19b**	>2500.00	96.78	25.831
**20a**	389.05	113.99	3.41	**20b**	**>2500.00**	**35.35**	**70.72**
**21a**	>500	∼174.98	∼2,86	**21b**	318.42	139.12	2.29
**22a**	500	95.28	5.25	**22b**	1881.91	64.17	29.33
**23a**	206.54	95.50	2.16	**23b**	>2500.00	140.54	17.79
**24a**	190.55	97.72	1.95	**24b**	144.94	61.70	2.35
**25a**	112.2	∼164.70	0.68	**25b**	755.61	285.43	2.65
**26a**	63.1	25.70	2.46	**26b**	230.94	156.89	1.47
**27a**	123.03	∼149.59	0.82	**27b**	425.30	148.01	2.87
**PYR**	11.37	1.79	6.34	**–**	**–**	**–**	**–**
**TRI**	336.20	20.93	16.07	**–**	**–**	**–**	**–**
**SDZ**	2161.72	4864.07	0.44	**–**	**–**	**–**	**–**

^a^Each value is expressed as the mean ± SD (*n* = 3). ^b^CC_30_ – the highest dilution of the samples to cause 30% or higher destruction of the L929 cells. ^c^IC_50_*_Tg_* – the concentration required to reduce *T. gondii* infected Hs27 cell growth by 50%. ^d^SI = selectivity index, a measure of efficacy, calculated by CC_30_/IC_50_*_Tg_*.

Reference drugs: PYR: pyrimethamine; TRI: trimethoprim; SDZ: sulfadiazine

Data for **1a**–**27a** were taken from Paneth et al.^39^^,^^40^

Bold values in Table 1 represent structural code number of the compounds under study. In this way, the compound numbering is bolded throughout the manuscript including those in Table 1.

Cytotoxic evaluation of the *s*-triazoles **1b**–**27b** on L929 cells revealed that, with some exception, the intramolecular cyclisation of thiosemicarbazides to the compounds with *s*-triazole scaffold results in significant reduction in cytotoxic effect, as evidenced by at least 4-fold increase in the CC_30_ values for the cyclic analogues compared to their linear precursors (e.g.**16a** *vs*. **16b**). For almost half of the *s*-triazoles tested, cytotoxic effect was seen at the concentration higher than 1000 µg/mL; six of them, **2b**, **13b**, **18b**, **19b**, **20b**, and **23b**, did not induce 30% or higher destruction of the L929 cells even at the highest test concentration of 2500.0 µg/mL. Except for **3b**, **9b**, **21b**, **24b**, and **26b**, all the *s*-triazoles were less cytotoxic than TRI and PYR. Additionally, those with CC_30_ higher than 2500.0 µg/mL were also less cytotoxic than SDZ. Although no direct structure-cytotoxic activity relationships were observed, it is highlight to note that mono methoxy substitution appears to prevent cytotoxic effect; for the structural isomers with *ortho* **18b**, *meta* **19b**, and *para* **20b** methoxy substitution cytotoxic effect was not seen up to the highest concentration tested.

### 3.3. Anti-toxoplasma gondii activity in vitro

**Figure 3.**
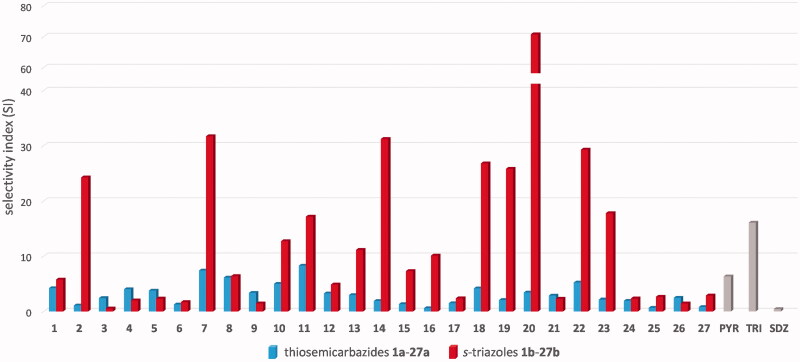
Selectivity index (SI) for the thiosemicarbazides **1a**–**27a** and the *s*-triazoles **1b**–**27b** calculated as the ratio of CC_30_ to IC_50_*_Tg_*. Reference drugs: PYR: pyrimethamine; TRI: trimethoprim; SDZ: sulfadiazine. Data for **1a**–**27a** were taken from Paneth et al.^39^^,^^40^

Next, we examined inhibitory effect of the *s*-triazoles **1b**–**27b** on *T. gondii* tachyzoites proliferation *in vitro*. SDZ, PYR, and TRI were used as positive control drugs. DMSO at a concentration of 0.1% was used as the negative control (data not shown). The IC_50_*_Tg_* results, defined as the inhibitory concentrations for 50% inhibition of *T. gondii* proliferation, are graphically presented in Figure 3. Details are reported in Table 1. The data for acyclic precursors **1a**–**27a** are also included for comparison^39^^,^^40^.

As presented in Table 1, although inhibitory effect of the *s*-triazoles **1b**–**27b** on *Toxoplasma* tachyzoites proliferation was generally weaker than those observed for the thiosemicarbazides **1a**–**27a**, a further calculation of the SI (calculated from CC_30_/IC_50_) showed that *s*-triazole scaffold is promising for anti-*Toxoplasma* activity, as indicated by a marked increase in their SI values compared to the linear precursors (e.g. **20a** *vs*. **20 b**, Figure 3). Among them, *para*-methoxy **20b** with SI of 70.72 is the most effective. It showed selectivity 160-fold more favourable than SDZ, 11-fold more favourable than PYR and 4-fold more favourable than TRI. Much more selective than all the three control drugs were also *meta*-F (**2b**, SI = 24.26), *ortho*-Br (**7b**, SI = 31.72), *meta*-I (**11b**, SI = 17.16), *ortho*-Me (**14b**, SI = 31.23), *ortho*-OMe (**18b**, SI = 26.82), *meta*-OMe (**19b**, SI = 25.83), *para*-OEt (**22b**, SI = 29.33), and *meta*-C(=O)Me (**18b**, SI = 17.79). The introduction of the second methoxy group to **20b** into the *meta* position gave **21b** with much lower selectivity (SI = 2.29) whereas four other *meta s*-triazoles (*meta*-F **2b**, *meta*-I **11**, *meta*-OMe **19b**, and *meta*-C(=O)CH_3_ **23b**) showed selectivity more favourable than all the three control drugs. This suggests that there is no direct relationship between the electronic effect and(or) substitution pattern at the N4 phenyl ring and specific anti-*Toxoplasma* action. All the *s*-triazoles showed better selectivity than SDZ. Five of them, *meta*-Br **8**, *ortho*-I **10**, 2,5-diF **13**, *meta*-Me **15b**, and *para*-Me **16b**, were additionally more selective than PYR but less than TRI.

### 3.4. In vitro haemolytic assay

The challenges of drug development for toxoplasmosis are multifactorial^43^ Apart from the challenge with achieving therapeutic concentrations in CNS, the main limiting factor in developing novel therapeutics is their cytotoxicity, particularly haematological, imposed by long treatment durations. In fact, PYR, a folic acid antagonist, is considered the most effective and standard component of current first-line therapy. At high doses or with long-term treatment, however, it can cause dose-related bone marrow suppression (e.g. megaloblastic anaemia, leukopoenia, granulocytopenia, and thrombocytopenia), resulting from folic acid deficiency^44^^,^^45^. Following SDZ, bone marrow suppression has also been reported in patients with the acquired immunodeficiency syndrome and toxoplasmic encephalitis^46^^,^^47^.

Apart from the bone marrow suppression-related anaemias, the RBCs can also be damaged during their circulation in the blood vessels. Drug-induced RBCs destruction process (haemolysis) can be caused by metabolic or immune factors^48^. Several mechanisms have been described to explain how drugs are able to induce immune-related haemolysis. These mechanisms include hapten mechanism, autoantibody production, membrane modification mechanism, and “innocent bystander” mechanism. In the metabolic pathway of drug-induced haemolysis, some drugs can oxidise the sulfhydryl groups of haemoglobin which leads to its denaturation and releasing from RBCs. In view of these facts, haemolytic activity evaluation seems to be essential at early stage of anti-*Toxoplasma* drug development. Good method for estimating haemotoxicity is haemolysis, characterised by RBCs rupture with release of haemoglobin. Increased plasma cell-free haemoglobin (CFH) can lead to haemoglobin toxicity (especially to vital organs, such as liver, kidney, and heart) or even mortality^49^. The haemolytic effect of our best anti-*Toxoplasma* candidate **20b** was assayed in human RBCs by spectrophotometric measurement of CFH level. Triton-X was used as positive control. As shown in Table 2, after treatment RBCs by the *s*-triazole **20b**, no statistically significant increase in the level of haemoglobin released to the medium was observed. It follows that the compound has no specific haemolytic activity at the concentration equal to its IC_50_*_Tg_*.

**Table 2.** Haemolytic effect of the *s*-triazole **20b.**

	Haemolysis (%)
*s*-triazole 20b [µg/mL]	
5 µg/mL	0.038 ± 0.007
10 µg/mL	0.104 ± 0.042
35 µg/mL	0.183 ± 0.086
untreated cells – negative control	0.041 ± 0.008
Triton-X (0.1%) – positive control	99.78 ± 0.34***

Each value is expressed as the mean ± SD (*n* = 3). Results were designated as statistically significant (ANOVA with post-hoc Tukey test) when *p* < 0.05 (*vs.* control group); ****p <  0.0001.

### 3.5. In vitro genotoxicity assay

**Figure 4.**
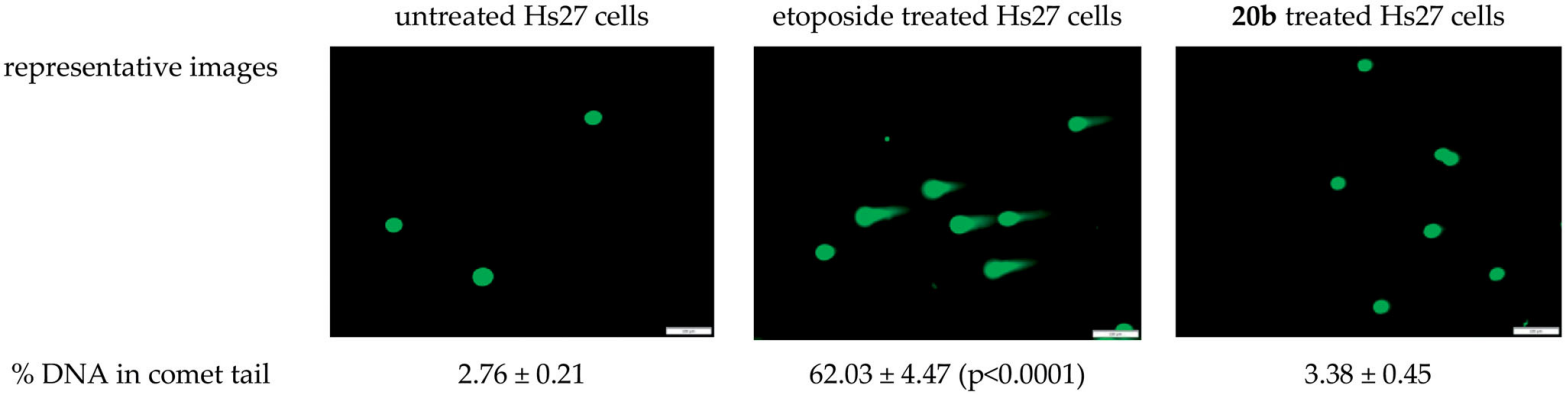
Genotoxic potential of the *s*-triazole **20b** evaluated in a single cell gel electrophoresis assay. The compounds was tested at the concentrations equal to its IC_50_*_Tg_*. Etoposide (positive control) was tested at 10 μg/mL. Statistical significance was calculated using ANOVA analysis followed by Dunnett’ *spost hoc* test (*vs*. untreated cells).

Since patients with toxoplasmosis usually need prolonged courses of treatment (e.g. congenitally infected neonates and immune-compromised patients*)*, another key issue at the early stage of anti-*Toxoplasma* drug development is genotoxic effect. Therefore, the alkaline single-cell gel electrophoresis (comet) assay was applied to assess *in vitro* genotoxic effect of *s*-triazole **20b** on human Hs27 cells. This assay sensitively detects DNA single- and double-strand breaks induced at the single cell level by chemical compounds^50^ and these DNA breaks are considered to be critical primary lesions leading to chromosomal aberrations, sister chromatid exchanges, and genotoxicity, if DNA damaged sites are not repaired. DNA breaks cause relaxation of supercoiled DNA fragments which then extend and migrate more rapidly under electrophoresis than intact DNA to form comet-like tails. The presence and the relative intensity of DNA in the comet-like tails reflect the DNA break frequency. The results of the assay shown that there is no induction of DNA damage in Hs-27 cells exposed to the *s*-triazole **20b** for 24 h (Figure 4). It follows that the compound at the concentration equal to its IC_50_*_Tg_* could be considered as a non-genotoxic scaffold.

### 3.6. Toxoplasma tachyzoites invasion and intracellular proliferation assays

**Figure 5.**
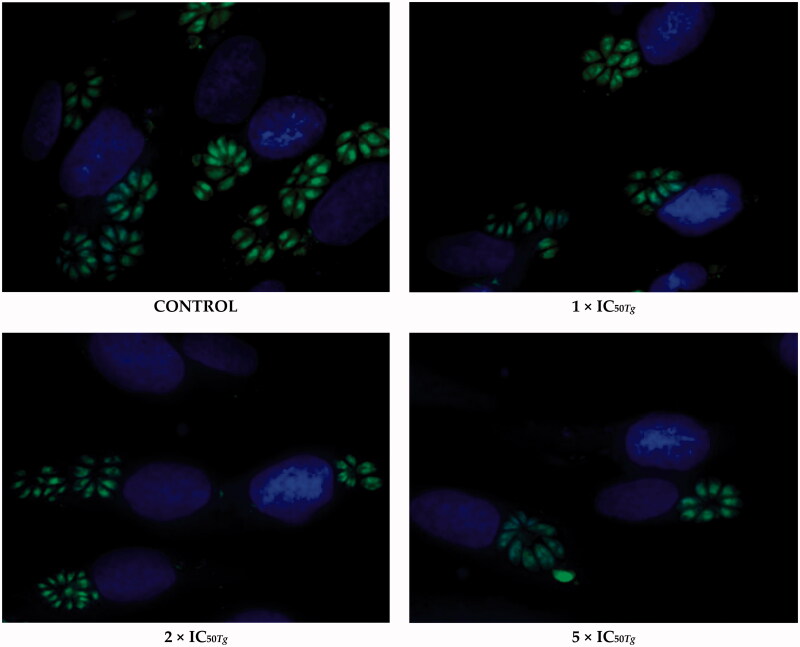
Representative images from *Toxoplasma* tachyzoites invasion assay. *T. gondii* tachyzoites were pre-incubated for 3 h with **20b** or culture medium alone (control group/untreated group). Then, the cell monolayers were rinsed and incubated in the treatment-free medium for a further 24 h. Untreated parasites (control group) were considered as 100% of invasion. *T. gondii* – green; cell nuclei – blue.

**Figure 6.**
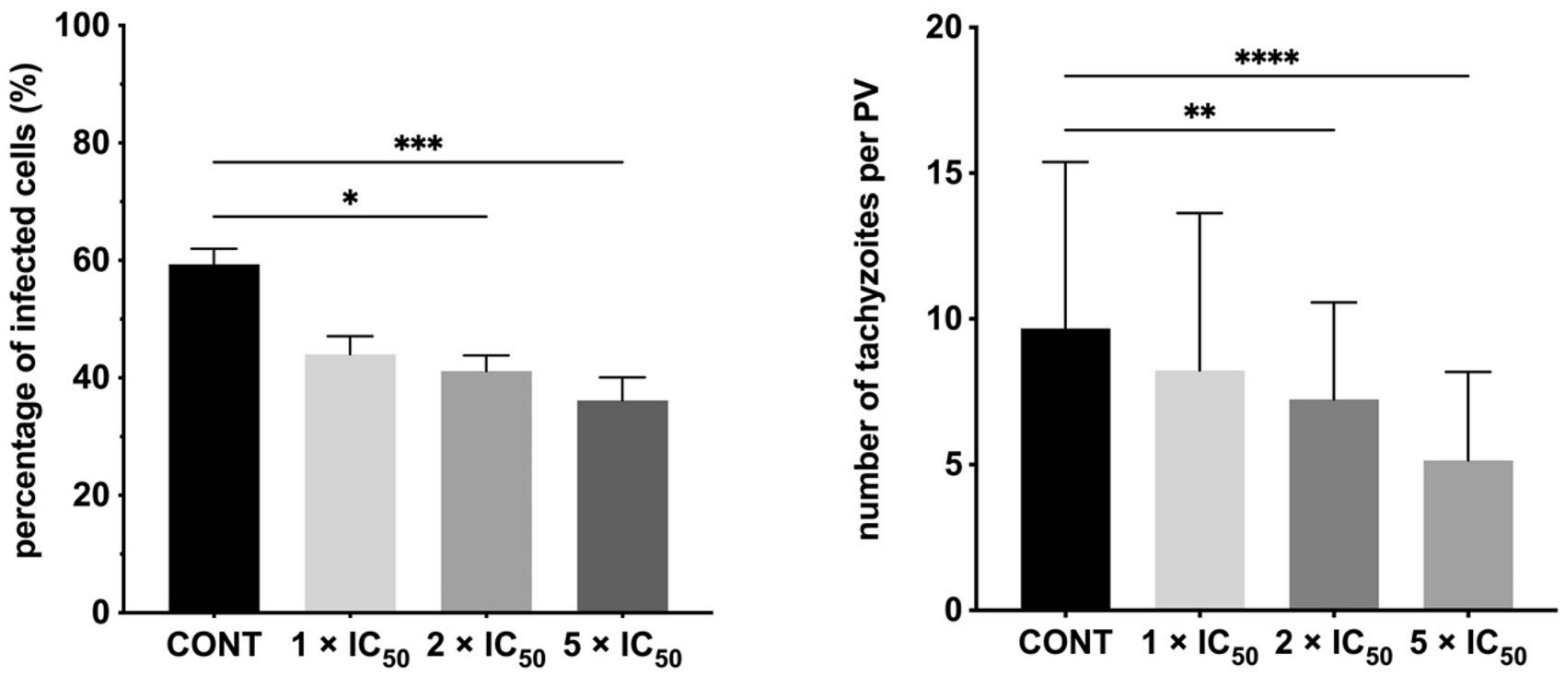
Influence of **20b** during *Toxoplasma* tachyzoites growth. *T. gondii* tachyzoites were pre-incubated for 3 h with **20b** or culture medium alone (control group/untreated group). Then, the cell monolayers were rinsed and incubated in the treatment-free medium for a further 24 h. (A) Percentage of infected cells. (B) Number of tachyzoites per PV. PV: parasitophorous vacuole; cont – control group (untreated parasites). Data shown are the combined averages of three independent experiments on triplicate chamber slides under the same conditions. Error bars indicate standard error of the mean. Values with statistically significant differences are labelled by brackets and asterisks as follows: ^****^p < 0.0001. Data were compared using a two-way ANOVA followed by Dunnett’s multiple comparisons test.

Although initial studies on triazole-based compounds have shown that these compounds can be effective and safe against *T. gondii* parasite^51^^,^^52^, to date there is little known on molecular mechanism underlying their anti-*Toxoplasma* mode of action. To address this missing knowledge, we have selected *s*-triazole **20b** to test its efficacy in tachyzoites invasion and subsequent intracellular proliferation. The success in the establishment of *T. gondii* infection requires the ability of the tachyzoites to attach and invade host cells. To evaluate whether **20b** is able to control the parasite invasion, we exposed host cells with tachyzoites for 3 h in the treatment-**20b** medium. Then, the cell monolayers were rinsed and incubated for a further 24 h in the treatment-free medium. Next, the percentage of infected cells and the number of tachyzoites per parasitophorous vacuole were measured. Hence, this assay differentiates between compounds that are able to act extracellularly (directly on the parasite) from those that can act intracellularly (within host cells). Concerning parasite invasion, our data suggest that the pre-treatment of *T. gondii* tachyzoites for 3 h with **20b** at the concentrations of 35.35 μg/mL (IC_50_*_Tg_*), 70.70 μg/mL (2 × IC_50_*_Tg_*), and 176.75 μg/mL (5 × IC_50_*_Tg_*) was not able to control the parasite invasion in the host cells. Specifically, data from microscopic analysis (Figure 5) followed by Dunnett’s multiple comparisons test after two-way ANOVA (Figure 6) show that **20b** did not cause significant reductions in percentage of infected cells, even at the highest test concentration of 176.75 μg/mL (5 × IC_50_*_Tg_*).

**Figure 7.**
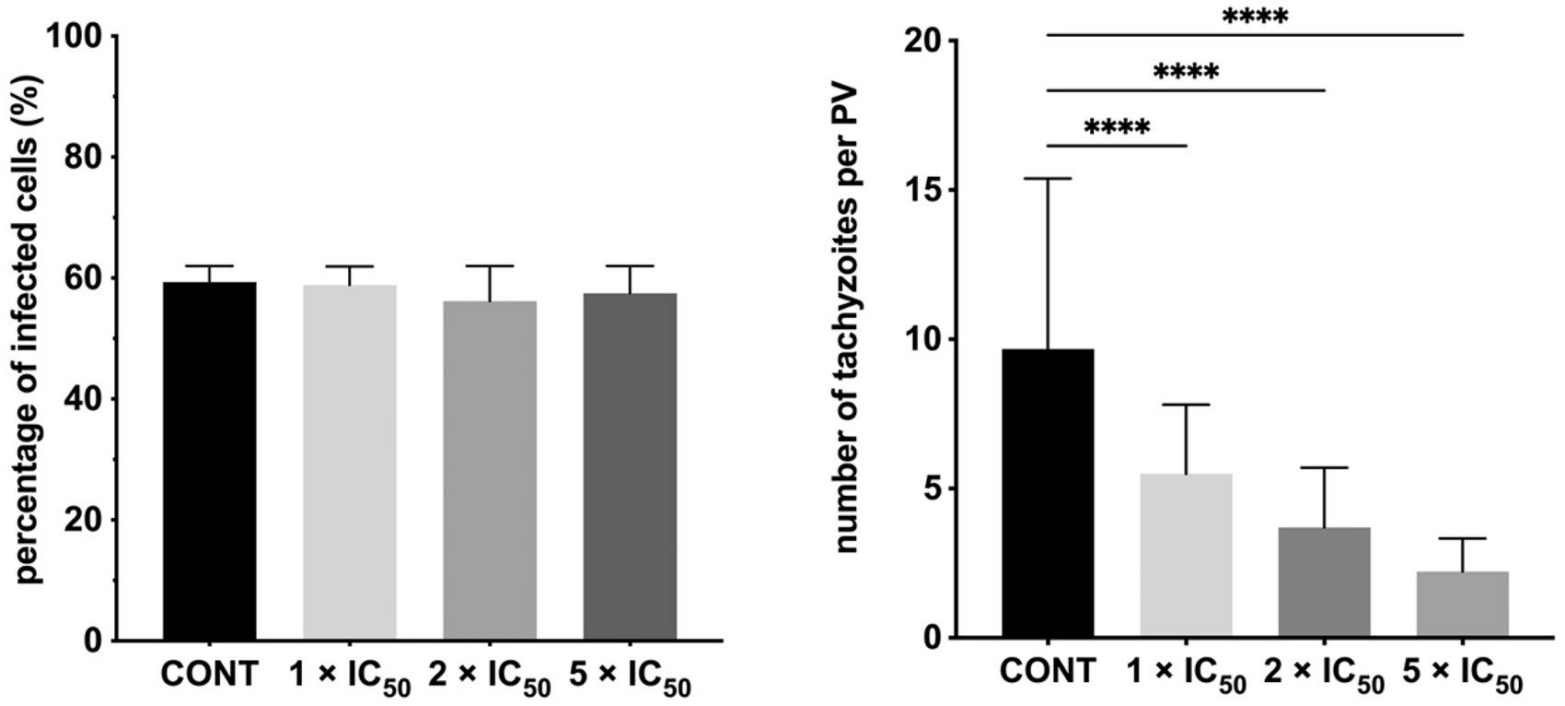
Influence of **20b** during intracellular *Toxoplasma* tachyzoites proliferation. *T. gondii* tachyzoites were exposed to the host cells for 3 h. Then, the cell monolayers were rinsed and incubated for a further 24 h in the treatment-**20b** medium or culture medium alone (control group/untreated group). (A) Percentage of infected cells. (B) Number of tachyzoites per PV. PV: parasitophorous vacuole; cont: control group (untreated parasites). Data shown are the combined averages of three independent experiments on triplicate chamber slides under the same conditions. Error bars indicate standard error of the mean. Values with statistically significant differences are labelled by brackets and asterisks as follows: ^****^p < 0.0001. Data were compared using a two-way ANOVA followed by Dunnett’s multiple comparisons test.

**Figure 8.**
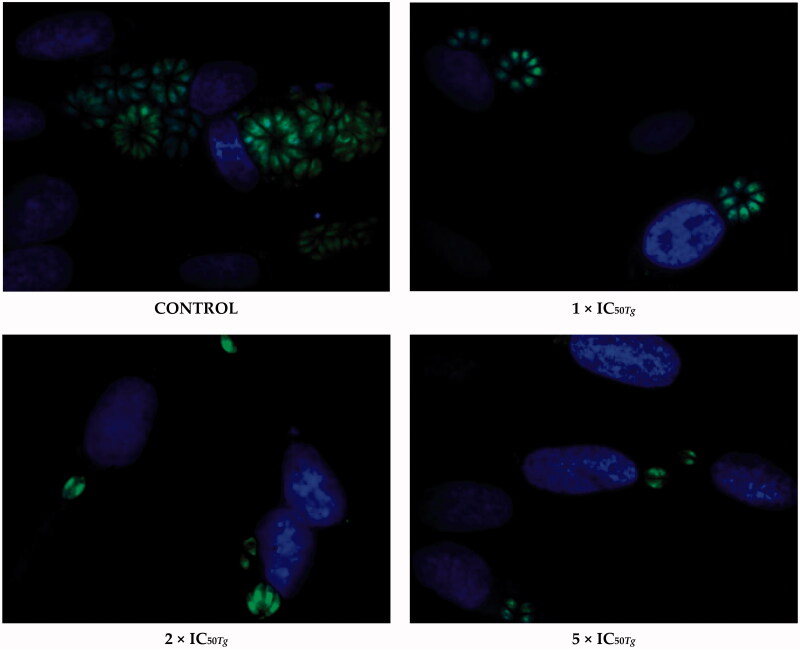
Representative images from *Toxoplasma* tachyzoites proliferation assay. *T. gondii* tachyzoites were exposed to the host cells for 3 h. Then, the cell monolayers were rinsed and incubated for a further 24 h in the treatment-**20b** medium or culture medium alone (control group/untreated group). Untreated parasites (control group) were considered as 100% of invasion. *T. gondii* – green, cell nuclei – blue.

Subsequently, to determine the impact of **20b** on the intracellular *T. gondii* proliferation, we have exposed host cells to tachyzoites for 3 h. Then, the cell monolayers were rinsed and incubated for a further 24 h in the treatment-**20b** medium. In parallel, we quantified the parasite proliferation at 24 h of treatment without **20b**; the results served as the baseline for comparison. As expected, after 24 h the percentage of infected cells in both experiments was still the same due to the fact tachyzoites had 3 h to enter the cell and to form the PV (Figure 7). However, for the cells incubated in the treatment-**20b** medium, the significant reduction in both size of the PV and number of intra vacuolar tachyzoites were observed, compared to the untreated group (control). It follows to conclude that **20b** is able to inhibit the parasite proliferation by impeding tachyzoites division and this effect is dose-dependent; the compound at the concentration of 176.75 μg/mL (5 × IC_50_*_Tg_*) proved to be more effective than at the concentration of 70.70 μg/mL (2 × IC_50_*_Tg_*); this concentration in turn was more effective than the lowest tested concentration of 35.35 μg/mL (IC_50_*_Tg_*). Representative images from proliferation assay are shown in Figure 8.

### 3.7. Anti-tyrosinase effect

As an intracellular parasite, *Toxoplasma* creates the PV during cell proliferation, which acts as an interface between the parasite and host cell cytoplasm and serves as a platform for modulating host cell functions to provide stable internal environment that support the parasite growth, protect it against host defences, and facilitate its replication and transmission. Reorganisation of the host’s spatial organelles and remodelling of the cytoskeleton around the PV are rapidly initiated after entry, and these processes favour the parasite to obtain nutrients and aminoacids, including arginine, histidine, valine, leucine, isoleucine, methionine, phenylalanine, tryptophan, and tyrosine, necessary for *Toxoplasma* growth^53^. Although the molecular mechanism of anti-*T. gondii* activity of triazole-based compounds remains unclear up to date, *in vitro* cytotoxicity and proliferation assays efforts show that host cell modulation by *T. gondii* is affected by **20b**, as evidenced by severe defects in PV formation. Recent studies on tyrosine metabolism in *Toxoplasma* tachyzoites confirmed that this amino acid is essential for the parasite growth and when access to exogenous tyrosine is limited it is impossible to efficiently form PV within invaded host cell^54^. Consistent with this, it is believed that compounds that are able to disrupt exogenous tyrosine uptake and(or) its metabolism in *Toxoplasma* have the potential to be developed into effective medicines against toxoplasmosis. Previously, we have reported for the first time that toxoplasmic modulation of tyrosine metabolism is involved in anti-*Toxoplasma* effect of thiosemicarbazide-based compounds^55^. Building on this observation, we thus tested the inhibitory action of *s*-triazole **20b** against tyrosinase (Tyr) activity. As shown in Figure S1 and Table S2 (Supplemental Material), respectively, weak inhibitory effects (< 10%) were recorded at the concentrations below 50 µM that reflects that **20b** targets another intracellular process important for *Toxoplasma* proliferation.

### 3.8. Pampa-BBB assay

Following primary infection, the tachyzoite disseminates widely in the organism *via* the bloodstream and lymphatic system. After passage of the BBB, they differentiate into cyst stages (bradyzoites), located predominantly in the neuronal cells of cerebral cortex, hippocampus, basal ganglia, and amygdala. This results in chronic, lifelong infection^56^^,^^57^. Chronic infection is usually asymptomatic and low rate of spontaneous reactivation is observed whereby bradyzoites differentiate back to tachyzoites. However, in patients with immune deficiency or those with prolonged treatments with immunosuppressants, reactivation of the infection can lead to lethal *Toxoplasma* encephalitis^23^^,^^58^. Additionally, chronic infection has been associated with development of neuropsychiatric disorders, including depression and schizophrenia^59–65^.

The fundamental problem blocking progress in the development of novel therapeutics for brain disorders is the BBB. According to literature data^66^, the transport of small molecules across the BBB is the exception rather than the rule, and 98% of all small molecules do not cross the BBB. It is generally considered that small molecules cross the BBB in pharmacologically-relevant concentration if their molecular mass is less than 400–500 Da and are able to form less than 8–10 hydrogen bonds with solvent water^67^. The *s*-triazole **20b**, with molecular weight 287.34, three H-bond acceptors, and two H-bond donors meets these criteria. Its ability to permeate through the BBB has also been confirmed experimentally, by the PAMPA-BBB assay. According to literature data^68^, high BBB permeation (CNS+) is expected for compounds with P_e_ > 5.19 (× 1 0 ^−6 ^cm·s ^−1^), whereas low BBB permeation (CNS−) is expected for compounds with P_e_ < 2.07 (× 1 0 ^−6 ^cm·s ^− 1^). The experimentally determined P_e_ value of 34.89 ± 2.73 (× 1 0 ^−6 ^cm·s ^− 1^), indicates that the *s*-triazole **20b** can be classified as centrally active.

### 3.9. Cytotoxic effect against human glioblastoma T98G cells

In 2020^69^, precedent epidemiologic studies have been published that provided the first prospective evidence linking *Toxoplasma* infection with risk of glioma. According to these data, the exposure to the tachyzoite stage of *Toxoplasma* infection may be aetiologically relevant in glioma. Although potential etiologic role for *Toxoplasma* in glioma risk is poorly understood thus far, the search for novel dual-action compounds, exerting both anti-*Toxoplasma* and anti-glioma activities, seems justified.

**Figure 9.**
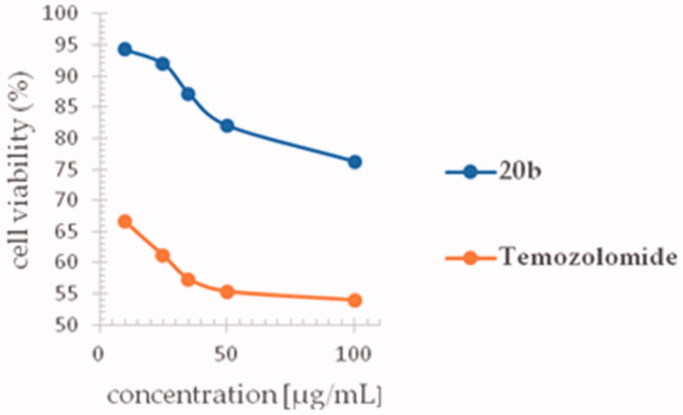
Cytotoxic effect of the *s*-triazole **20b** and temozolomide against human glioblastoma T98G cell line. Cell viability was determined by MTT assay after the treatment with various concentrations of the compounds (100, 50, 35, 25, and 10 µg/mL) for 24 h.

To evaluate anti-glioma potency of *s*-triazole **20b**, human glioblastoma T98G cell line was selected as the glioma model. According to the MTT assay results presented in Figure 9, treatment the T98G cells with **20b** reduced cell viability in a similar dose-dependent manner (100 > 50 > 35 > 25 > 10 µg/mL) to temozolomide (control drug). Compared to temozolomide, however, *s*-triazole **20b** produced weaker inhibitory effect (Table 3); the highest test concentration of 100 µg/mL reduced cell viability by ∼24% (*vs*. ∼46% for temozolomide) whereas the lowest one (10 µg/mL) by ∼6% (*vs.* ∼33% for temozolomide) after 24 h of treatment.

**Table 3.** Cytotoxic effect of the *s*-triazole against human glioblastoma T98G cells.

	Viability of T98G cells (% of control ± SD)	
Concentration [µg/mL]	*s*-triazole **20b**	Temozolomide
10	94.25 ± 2.06	66.56 ± 1.07
25	92.00 ± 3.27	61.11 ± 0.45
35	87.15 ± 0.55	57.32 ± 2.25
50	82.06 ± 1.73	55.34 ± 1.81
100	76.26 ± 0.74	53.95 ± 0.33

Each value is expressed as the mean ± SD (*n* = 3).

## 4. Conclusion

In summary, a series of novel *s*-triazoles were designed, synthesised, and evaluated as potential anti-*Toxoplasma* agents. The best of them (**2b**, **7b**, **11b**, **14b**, **18b**, **19b**, **20b**, **22b**, and **18b**) were more selective than all three control drugs; SDZ, PYR, and TRI. Among them the most potent was *para*-methoxy compound **20b** with selectivity 160-fold more favourable than SDZ, 11-fold more favourable than PYR, and 4-fold more favourable than TRI. In cellular level, **20b** at the concentration equal to its IC_50_*_Tg_* showed no genotoxic effect on Hs27 cells and no specific haemolytic changes in RBCs. Moreover, **20b** could cross the BBB, which is a critical factor linked with an ideal anti-*Toxoplasma* drug development. Taken together, *s*-triazole **20b** can be considered a candidate for the preclinical stage as well as an interesting lead structure in the search for novel anti-*Toxoplasma* agents.

## Supplementary Material

Supplemental Material
