# Supplementary material for: Inhibition of Toxoplasma gondii by 1,2,4-triazole-based compounds: marked improvement in selectivity relative to the standard therapy pyrimethamine and sulfadiazine
Source: J Enzyme Inhib Med Chem. 2022 Sep 27;37(1):2621–34. doi: 10.1080/14756366.2022.2112576 (PMC9518248; doi:10.1080/14756366.2022.2112576)

Inhibition of *Toxoplasma gondii* by 1,2,4-triazole-based compounds: marked improvement in selectivity relative to the standard therapy pyrimethamine and sulfadiazine

Lidia Węglińska,[1] Adrian Bekier,[2] Nazar Trotsko,[1] Barbara Kaproń,[3] Tomasz Plech,[4] Katarzyna Dzitko,[2] Agata Paneth[1]*

[1]*Department of Organic Chemistry, Medical University of Lublin, Chodźki 4a, 20-093 Lublin, Poland*

[2]*Department of Molecular Microbiology, Faculty of Biology and Environmental Protection, University of Lodz, 90-237 Lodz, Poland*

[3]*Department of Clinical Genetics, Medical University of Lublin, Radziwiłłowska 11, 20-080 Lublin, Poland*

[4]*Department of Pharmacology, Medical University of Lublin, Radziwiłłowska 11, 20-080 Lublin, Poland*

*Author to whom correspondence should be addressed: agata.paneth@umlub.pl

Supplemental material

Table S1. *In silico* ADME prediction of the *s*-triazoles **1b-27b**……………………….………...3

Figure S1. Inhibitory effect of the *s*-triazole **20b** on tyrosinase (Tyr) activity. Error bars indicate standard error of the mean……………………………………………………………….…7

Table S2. Inhibitory effect of the *s*-triazole **20b** on tyrosinase activity……………………….7

NMR spectra………………………………………………………………………………..8

Table S1. *In silico* ADME prediction of the *s*-triazoles **1b-27b**.

Compd.	water solubility[1]	Log *P*[2]	pharmacokinetics[3]	drug-likeness[4]	medicinal chemistry friendliness[5]
**1b**	soluble	2.52	GI absorption: *high*<br>P-gp substrate: *no*<br>CYP1A2 inhibitor: *yes*<br>CYP2C19 inhibitor: *no*<br>CYP2C9 inhibitor: *no*<br>CYP2D6 inhibitor: *no*<br>CYP3A4 inhibitor: *no*	rule-based filters: *yes*<br>bioavailability score: *0.55*	PAINS: *0 alert*<br>leadlikeness: *yes*
**2b**	soluble	2.53	GI absorption: *high*<br>P-gp substrate: *no*<br>CYP1A2 inhibitor: *yes*<br>CYP2C19 inhibitor: *no*<br>CYP2C9 inhibitor: *no*<br>CYP2D6 inhibitor: *no*<br>CYP3A4 inhibitor: *no*	rule-based filters: *yes*<br>bioavailability score: *0.55*	PAINS: *0 alert*<br>leadlikeness: *yes*
**3b**	soluble	2.54	GI absorption: *high*<br>P-gp substrate: *no*<br>CYP1A2 inhibitor: *yes*<br>CYP2C19 inhibitor: *no*<br>CYP2C9 inhibitor: *no*<br>CYP2D6 inhibitor: *no*<br>CYP3A4 inhibitor: *no*	rule-based filters: *yes*<br>bioavailability score: *0.55*	PAINS: *0 alert*<br>leadlikeness: *yes*
**4b**	soluble	2.72	GI absorption: *high*<br>P-gp substrate: *no*<br>CYP1A2 inhibitor: *yes*<br>CYP2C19 inhibitor: *yes*<br>CYP2C9 inhibitor: *no*<br>CYP2D6 inhibitor: *no*<br>CYP3A4 inhibitor: *no*	rule-based filters: *yes*<br>bioavailability score: *0.55*	PAINS: *0 alert*<br>leadlikeness: *yes*
**5b**	soluble	2.75	GI absorption: *high*<br>P-gp substrate: *no*<br>CYP1A2 inhibitor: *yes*<br>CYP2C19 inhibitor:*yes*<br>CYP2C9 inhibitor: *no*<br>CYP2D6 inhibitor: *no*<br>CYP3A4 inhibitor: *no*	rule-based filters: *yes*<br>bioavailability score: *0.55*	PAINS: *0 alert*<br>leadlikeness: *yes*
**6b**	soluble	2.76	GI absorption: *high*<br>P-gp substrate: *no*<br>CYP1A2 inhibitor: *yes*<br>CYP2C19 inhibitor: *yes*<br>CYP2C9 inhibitor: *no*<br>CYP2D6 inhibitor: *no*<br>CYP3A4 inhibitor: *no*	rule-based filters: *yes*<br>bioavailability score: *0.55*	PAINS: *0 alert*<br>leadlikeness: *yes*
**7b**	moderately soluble	2.81	GI absorption: *high*<br>P-gp substrate: *no*<br>CYP1A2 inhibitor: *yes*<br>CYP2C19 inhibitor: *yes*<br>CYP2C9 inhibitor: *no*<br>CYP2D6 inhibitor: *no*<br>CYP3A4 inhibitor: *no*	rule-based filters: *yes*<br>bioavailability score: *0.55*	PAINS: *0 alert*<br>structural alert: *thiocarbonyl group*
**8b**	moderately soluble	2.84	GI absorption: *high*<br>P-gp substrate: *no*<br>CYP1A2 inhibitor: *yes*<br>CYP2C19 inhibitor: *yes*<br>CYP2C9 inhibitor: *no*<br>CYP2D6 inhibitor: *no*<br>CYP3A4 inhibitor: *no*	rule-based filters: *yes*<br>bioavailability score: *0.55*	PAINS: *0 alert*<br>leadlikeness: *yes*
**9b**	moderately soluble	2.85	GI absorption: *high*<br>P-gp substrate: *no*	rule-based filters: *yes*<br>bioavailability score: *0.55*	PAINS: *0 alert*<br>leadlikeness: *yes*

			CYP1A2 inhibitor: *yes*<br>CYP2C19 inhibitor: *yes*<br>CYP2C9 inhibitor: *no*<br>CYP2D6 inhibitor: *no*<br>CYP3A4 inhibitor: *no*		
**10b**	moderately soluble	2.85	GI absorption: *high*<br>P-gp substrate: *no*<br>CYP1A2 inhibitor: *yes*<br>CYP2C19 inhibitor: *yes*<br>CYP2C9 inhibitor: *no*<br>CYP2D6 inhibitor: *no*<br>CYP3A4 inhibitor: *no*	rule-based filters: *yes*<br>bioavailability score: *0.55*	PAINS: *0 alert*<br>leadlikeness: *no; 1violation: MW >350*
**11b**	moderately soluble	2.94	GI absorption: *high*<br>P-gp substrate: *no*<br>CYP1A2 inhibitor: *yes*<br>CYP2C19 inhibitor: *yes*<br>CYP2C9 inhibitor:*yes*<br>CYP2D6 inhibitor: *no*<br>CYP3A4 inhibitor: *yes*	rule-based filters: *yes*<br>bioavailability score: *0.55*	PAINS: *0 alert*<br>leadlikeness: *no; 1violation: MW >350*
**12b**	moderately soluble	2.90	GI absorption: *high*<br>P-gp substrate: *no*<br>CYP1A2 inhibitor: *yes*<br>CYP2C19 inhibitor: *yes*<br>CYP2C9 inhibitor: *yes*<br>CYP2D6 inhibitor: *no*<br>CYP3A4 inhibitor: *no*	rule-based filters: *yes*<br>bioavailability score: *0.55*	PAINS: *0 alert*<br>leadlikeness: *no; 1violation: MW >350*
**13b**	soluble	2.82	GI absorption: *high*<br>P-gp substrate: *no*<br>CYP1A2 inhibitor: *yes*<br>CYP2C19 inhibitor:*no*<br>CYP2C9 inhibitor:*no*<br>CYP2D6 inhibitor: *no*<br>CYP3A4 inhibitor: *no*	rule-based filters: *yes*<br>bioavailability score: *0.55*	PAINS: *0 alert*<br>leadlikeness: *yes*
**14b**	soluble	2.57	GI absorption: *high*<br>P-gp substrate: *no*<br>CYP1A2 inhibitor: *yes*<br>CYP2C19 inhibitor: *yes*<br>CYP2C9 inhibitor: *no*<br>CYP2D6 inhibitor: *no*<br>CYP3A4 inhibitor: *yes*	rule-based filters: *yes*<br>bioavailability score: *0.55*	PAINS: *0 alert*<br>leadlikeness: *yes*
**15b**	soluble	2.56	GI absorption: *high*<br>P-gp substrate: *no*<br>CYP1A2 inhibitor: *yes*<br>CYP2C19 inhibitor: *yes*<br>CYP2C9 inhibitor: *no*<br>CYP2D6 inhibitor: *no*<br>CYP3A4 inhibitor: *yes*	rule-based filters: *yes*<br>bioavailability score: *0.55*	PAINS: *0 alert*<br>leadlikeness: *yes*
**16b**	soluble	2.57	GI absorption: *high*<br>P-gp substrate: *no*<br>CYP1A2 inhibitor: *yes*<br>CYP2C19 inhibitor: *yes*<br>CYP2C9 inhibitor: *no*<br>CYP2D6 inhibitor: *no*<br>CYP3A4 inhibitor: *yes*	rule-based filters: *yes*<br>bioavailability score: *0.55*	PAINS: *0 alert*<br>leadlikeness: *yes*
**17b**	moderately soluble	3.15	GI absorption: *high*<br>P-gp substrate: *no*<br>CYP1A2 inhibitor: *yes*<br>CYP2C19 inhibitor: *yes*<br>CYP2C9 inhibitor: *yes*<br>CYP2D6 inhibitor: *no*<br>CYP3A4 inhibitor: *yes*	rule-based filters: *yes*<br>bioavailability score: *0.55*	PAINS: *0 alert*<br>leadlikeness: *yes*
**18b**	soluble	2.14	GI absorption: *high*	rule-based filters: *yes*	PAINS: *0 alert*

			P-gp substrate: *no*<br>CYP1A2 inhibitor: *yes*<br>CYP2C19 inhibitor: *no*<br>CYP2C9 inhibitor: *no*<br>CYP2D6 inhibitor: *no*<br>CYP3A4 inhibitor: *yes*	bioavailability score: *0.55*	leadlikeness: *yes*
**19b**	soluble	2.15	GI absorption: *high*<br>P-gp substrate: *no*<br>CYP1A2 inhibitor: *yes*<br>CYP2C19 inhibitor: *no*<br>CYP2C9 inhibitor: *no*<br>CYP2D6 inhibitor: *no*<br>CYP3A4 inhibitor: *yes*	rule-based filters: *yes*<br>bioavailability score: *0.55*	PAINS: *0 alert*<br>leadlikeness: *yes*
**20b**	soluble	2.15	GI absorption: *high*<br>P-gp substrate: *no*<br>CYP1A2 inhibitor: *yes*<br>CYP2C19 inhibitor: *no*<br>CYP2C9 inhibitor: *no*<br>CYP2D6 inhibitor: *no*<br>CYP3A4 inhibitor: *yes*	rule-based filters: *yes*<br>bioavailability score: *0.55*	PAINS: *0 alert*<br>leadlikeness: *yes*
**21b**	soluble	2.13	GI absorption: *high*<br>P-gp substrate: *no*<br>CYP1A2 inhibitor: *yes*<br>CYP2C19 inhibitor: *no*<br>CYP2C9 inhibitor: *yes*<br>CYP2D6 inhibitor: *no*<br>CYP3A4 inhibitor: *yes*	rule-based filters: *yes*<br>bioavailability score: *0.55*	PAINS: *0 alert*<br>leadlikeness: *yes*
**22b**	soluble	2.48	GI absorption: *high*<br>P-gp substrate: *no*<br>CYP1A2 inhibitor: *yes*<br>CYP2C19 inhibitor:*yes*<br>CYP2C9 inhibitor:*yes*<br>CYP2D6 inhibitor: *no*<br>CYP3A4 inhibitor: *yes*	rule-based filters: *yes*<br>bioavailability score: *0.55*	PAINS: *0 alert*<br>leadlikeness: *yes*
**23b**	soluble	2.12	GI absorption: *high*<br>P-gp substrate: *no*<br>CYP1A2 inhibitor: *yes*<br>CYP2C19 inhibitor: *no*<br>CYP2C9 inhibitor: *no*<br>CYP2D6 inhibitor: *no*<br>CYP3A4 inhibitor: *yes*	rule-based filters: *yes*<br>bioavailability score: *0.55*	PAINS: *0 alert*<br>leadlikeness: *yes*
**24b**	moderately soluble	4.01	GI absorption: *high*<br>P-gp substrate: *no*<br>CYP1A2 inhibitor: *yes*<br>CYP2C19 inhibitor: *yes*<br>CYP2C9 inhibitor: *yes*<br>CYP2D6 inhibitor: *no*<br>CYP3A4 inhibitor: *yes*	rule-based filters: *yes*<br>bioavailability score: *0.55*	PAINS: *0 alert*<br>leadlikeness: *no; 1 violation: XLOGP3>3.5*
**25b**	soluble	2.14	GI absorption: *high*<br>P-gp substrate: *no*<br>CYP1A2 inhibitor: *yes*<br>CYP2C19 inhibitor: *no*<br>CYP2C9 inhibitor: *yes*<br>CYP2D6 inhibitor: *no*<br>CYP3A4 inhibitor: *yes*	rule-based filters: *yes*<br>bioavailability score: *0.55*	PAINS: *1 alert; aminoaryl ring*<br>leadlikeness: *yes*
**26b**	soluble	2.81	GI absorption: *high*<br>P-gp substrate: *no*<br>CYP1A2 inhibitor: *yes*<br>CYP2C19 inhibitor: *yes*<br>CYP2C9 inhibitor: *yes*<br>CYP2D6 inhibitor: *no*<br>CYP3A4 inhibitor: *yes*	rule-based filters: *yes*<br>bioavailability score: *0.55*	PAINS: *1 alert; aminoaryl ring*<br>leadlikeness: *yes*

**27b**	soluble	2.32	GI absorption: *high*<br>P-gp substrate: *no*<br>CYP1A2 inhibitor: *no*<br>CYP2C19 inhibitor: *yes*<br>CYP2C9 inhibitor: *no*<br>CYP2D6 inhibitor: *no*<br>CYP3A4 inhibitor: *no*	rule-based filters: *yes*<br>bioavailability score: *0.55*	PAINS: *0 alert*<br>leadlikeness: *yes*

[1]**Log *S*** calculated with ESOL model. Scale: insoluble < -10 < poorly < -6 <moderately < -4 < soluble < -2 < very < 0 <highly. [2]consensus log $P_{o/w}$: the arithmetic mean of the values predicted by the five models (XLOGP3, WLOGP, MLOGP, SILICOS-IT, iLOGP).[3]**GI** – gastrointestinal, **P-gp**– P-protein; [4]drug-likeness assesses qualitatively the chance for a molecule to become an oral drug with respect to bioavailability. **Rule-based filters**: Lipinski, Ghose, Veber, Egan, Muegge filers. **Bioavailability score**: the probability of a compound to have at least 10% oral bioavailability in rat or measurable Caco-2 permeability. This score defines four classes of compounds with probabilities of 11%, 17%, 56% or 85%. [5]**PAINS**: structural fragments found in the molecule under evaluation yielding false positive biological output. **Leadlikeness**: a molecular entity suitable for optimization.

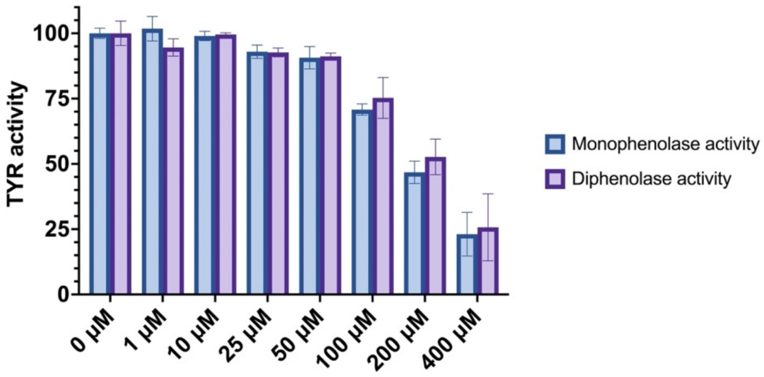

Figure S1. Inhibitory effect of the *s*-triazole **20b** on tyrosinase (Tyr) activity. Error bars indicate standard error of the mean. For details see Table S2

Table S2. Inhibitory effect of the *s*-triazole **20b** on tyrosinase activity

conc. [µM]	Monophenolase activity [%] ± SD	Diphenolase activity [%] ± SD
**0**	100.00 ± 1.95	100.0 ± 4.7
**1**	101.76 ± 4.69	94.6 ± 3.3
**10**	99.01 ± 1.79	99.6 ± 0.7
**25**	92.97 ± 2.54	92.7 ± 1.7
**50**	90.66 ± 4.29	91.2 ± 1.2
**100**	70.81 ± 2.18	75.3 ± 7.8
**200**	46.79 ± 4.31	52.7 ± 6.8
**400**	23.09 ± 8.35	25.7 ± 12.8

Each value is expressed as the mean ± SD (n = 3)

Compound **2b**

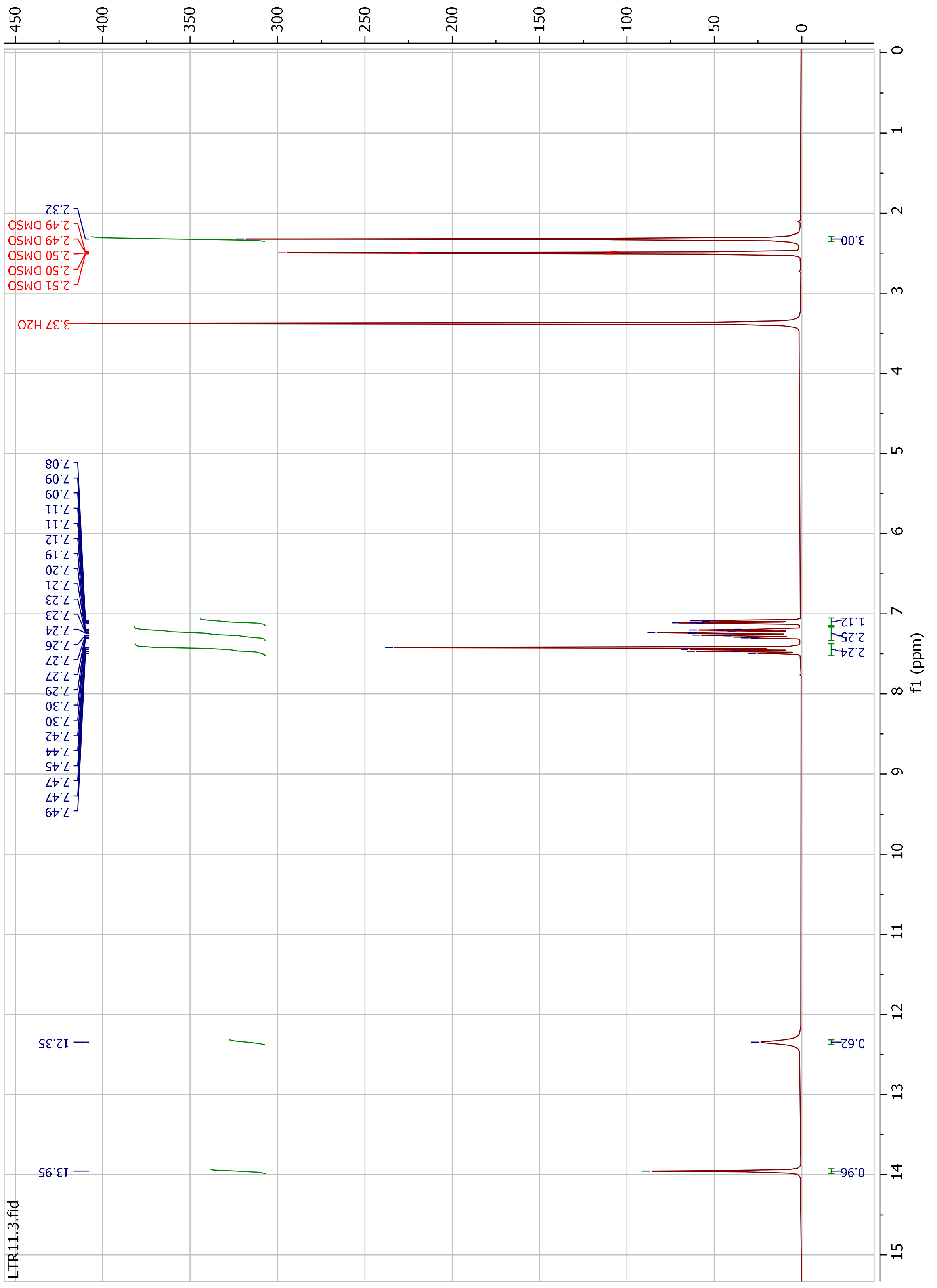
2.32
2.49 DMSO
2.49 DMSO
2.50 DMSO
2.50 DMSO
2.51 DMSO
3.37 H2O
7.08
7.09
7.09
7.11
7.11
7.12
7.19
7.20
7.21
7.23
7.23
7.24
7.26
7.27
7.27
7.29
7.30
7.30
7.42
7.44
7.45
7.47
7.47
7.49
12.35
13.95
3.00
1.12
2.25
2.24
0.62
0.96
f1 (ppm)
LTR11.3.fid

Compound **4b**

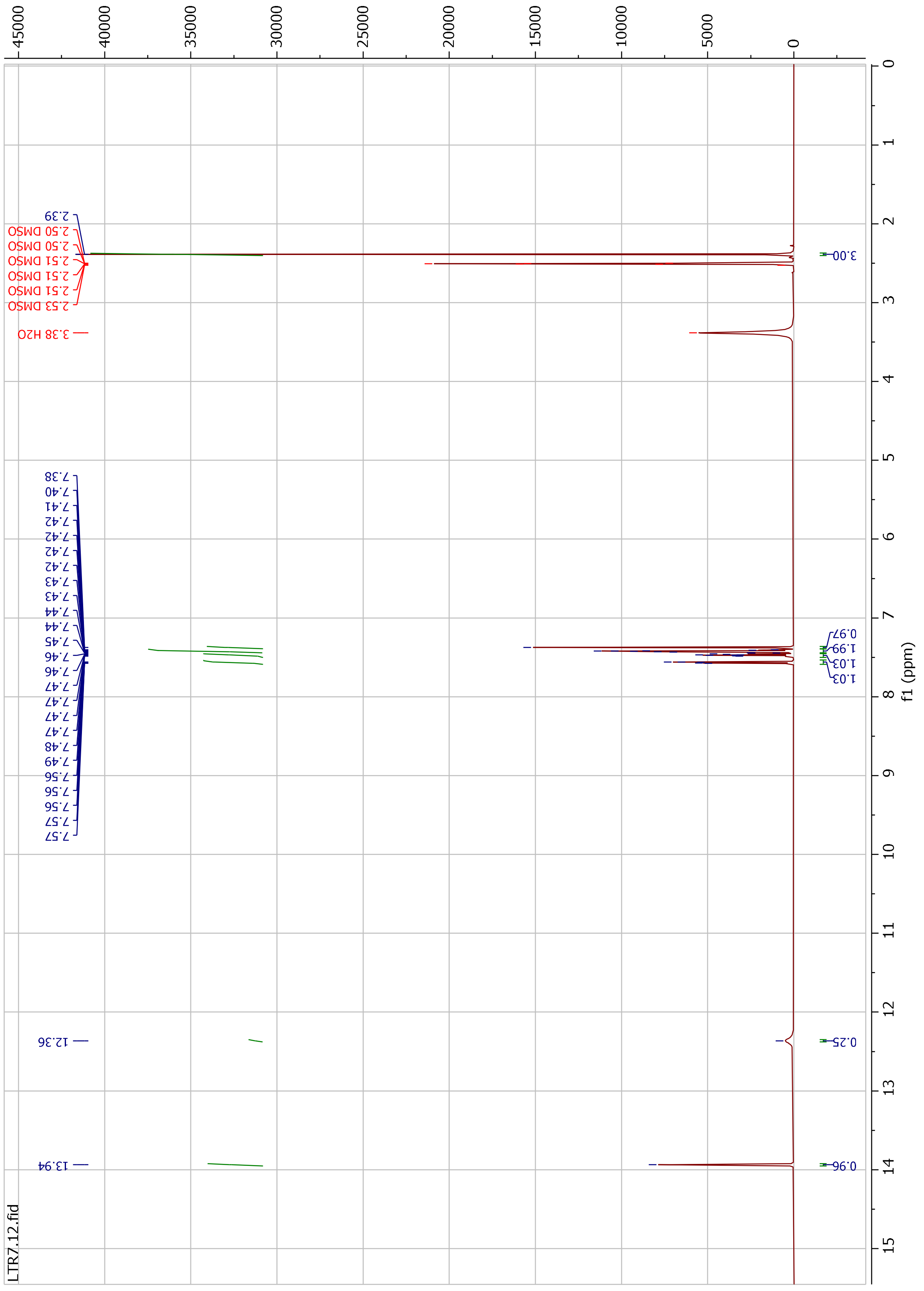
LTR7.12.fid
f1 (ppm)
2.39
2.50 DMSO
2.50 DMSO
2.51 DMSO
2.51 DMSO
2.51 DMSO
2.53 DMSO
3.38 H2O
7.38
7.40
7.41
7.42
7.42
7.42
7.42
7.43
7.43
7.44
7.44
7.45
7.46
7.46
7.47
7.47
7.47
7.47
7.48
7.49
7.56
7.56
7.56
7.57
7.57
12.36
13.94
3.00
0.97
1.99
1.03
1.03
0.25
0.96

Compound **5b**

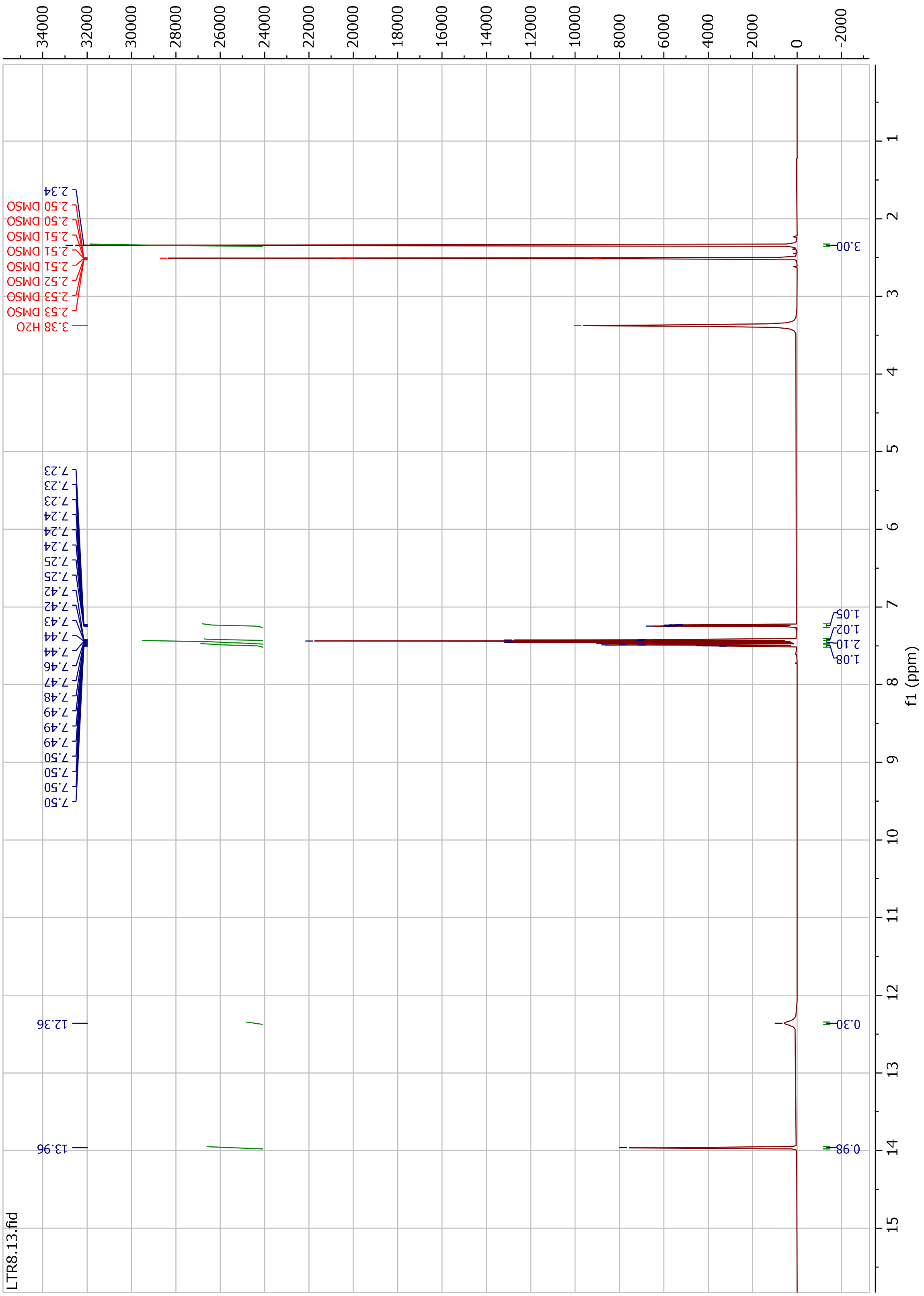

LTR8.13.fid
f1 (ppm)
2.34
2.50 DMSO
2.50 DMSO
2.51 DMSO
2.51 DMSO
2.51 DMSO
2.52 DMSO
2.53 DMSO
2.53 DMSO
3.38 H2O
12.36
13.96
3.00
1.05
1.02
2.10
1.08
0.30
0.98

Compound **6b**

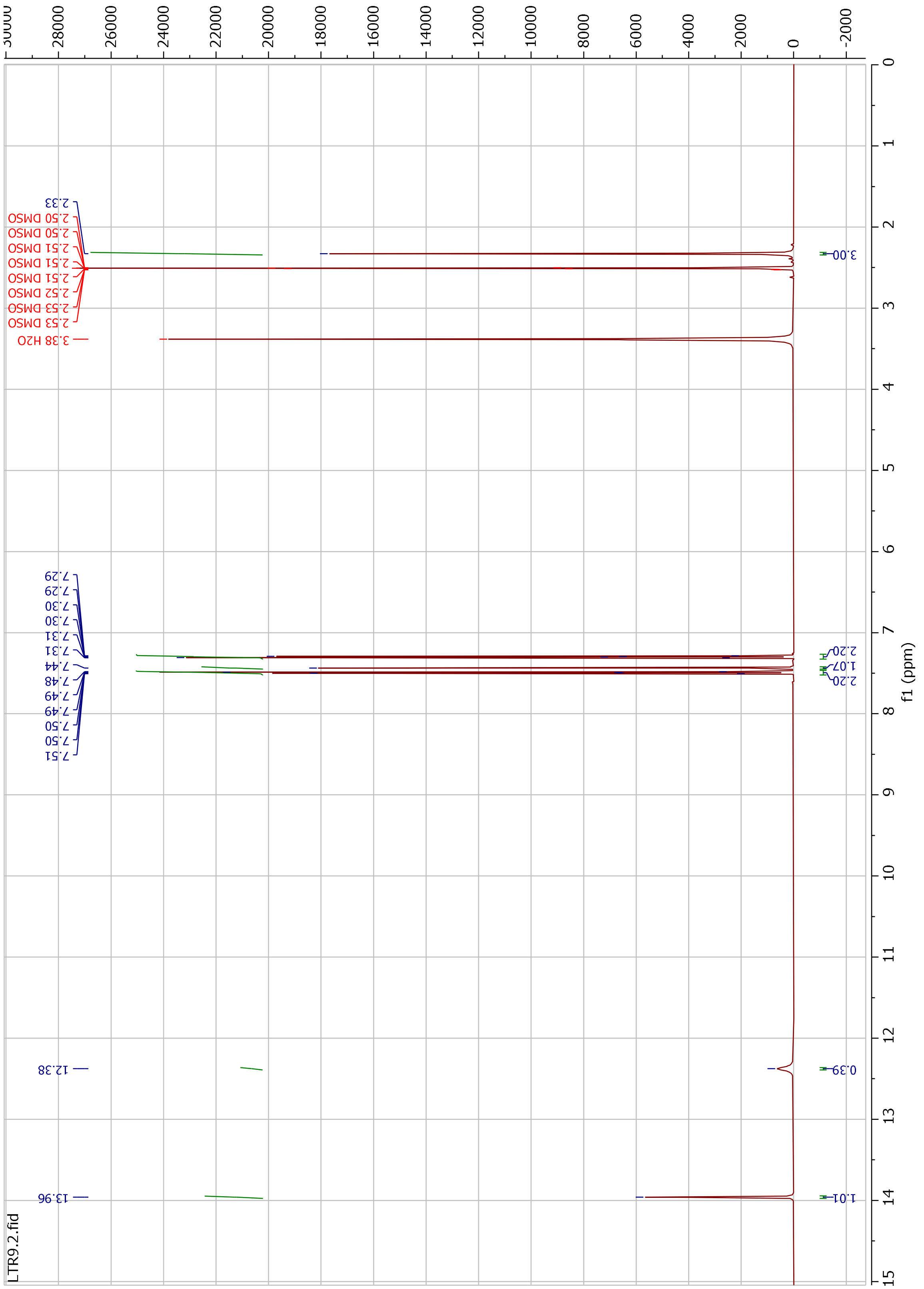

2.33
2.50 DMSO
2.51 DMSO
2.51 DMSO
2.51 DMSO
2.52 DMSO
2.53 DMSO
2.53 DMSO
3.38 H2O
7.29
7.29
7.30
7.30
7.31
7.31
7.44
7.48
7.49
7.49
7.50
7.50
7.51
12.38
13.96
3.00
2.20
1.07
2.20
0.39
1.01
f1 (ppm)
LTR9.2.fid

Compound **7b**

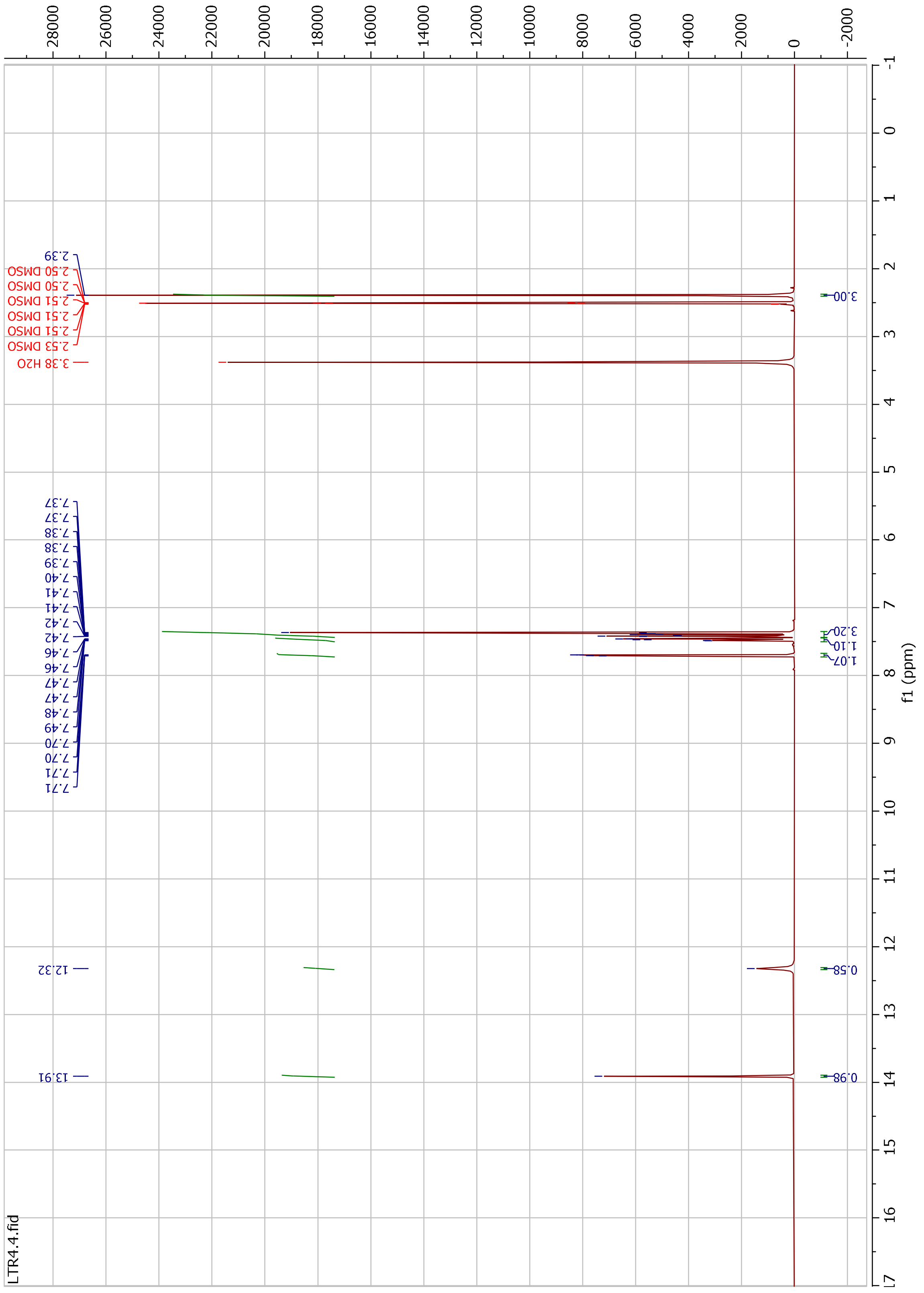

2.39
2.50 DMSO
2.50 DMSO
2.51 DMSO
2.51 DMSO
2.51 DMSO
2.53 DMSO
3.38 H2O
7.37
7.37
7.38
7.38
7.39
7.40
7.41
7.41
7.42
7.42
7.46
7.46
7.47
7.47
7.48
7.49
7.70
7.70
7.71
7.71
12.32
13.91
3.00
3.20
1.10
1.07
0.58
0.98
f1 (ppm)
LTR4.4.fid

Compound **8b**

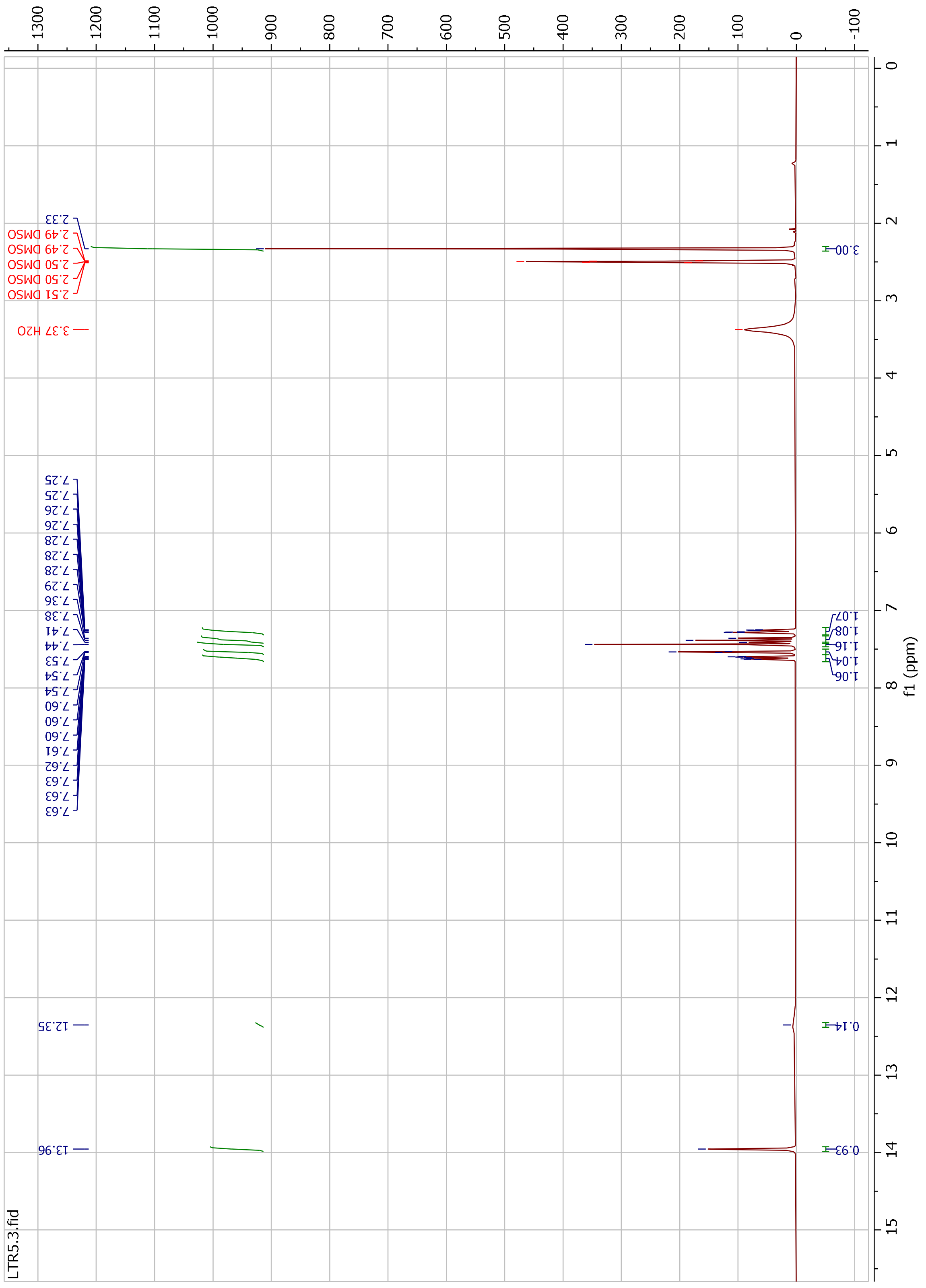

LTR5.3.fid
f1 (ppm)
2.33
2.49 DMSO
2.49 DMSO
2.50 DMSO
2.50 DMSO
2.51 DMSO
3.37 H2O
7.25
7.25
7.26
7.26
7.28
7.28
7.28
7.29
7.36
7.38
7.41
7.44
7.53
7.54
7.54
7.60
7.60
7.60
7.61
7.62
7.63
7.63
7.63
12.35
13.96
3.00
1.07
1.08
1.16
1.04
1.06
0.14
0.93

Compound **9b**

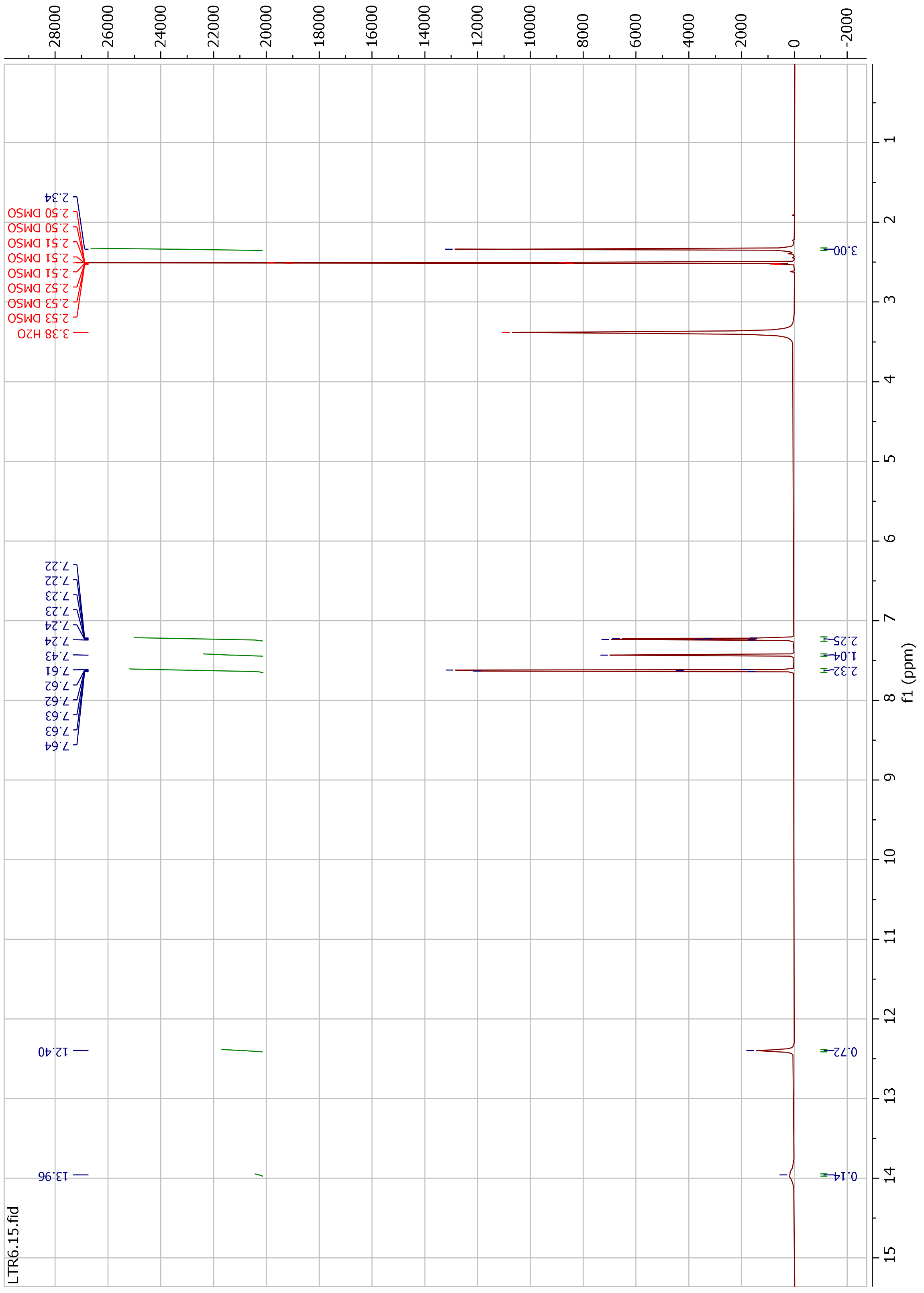
LTR6.15.fid
f1 (ppm)
2.34
2.50 DMSO
2.50 DMSO
2.51 DMSO
2.51 DMSO
2.51 DMSO
2.52 DMSO
2.53 DMSO
2.53 DMSO
3.38 H2O
7.22
7.22
7.23
7.23
7.24
7.24
7.43
7.61
7.62
7.62
7.63
7.63
7.64
12.40
13.96
3.00
2.25
1.04
2.32
0.72
0.14

Compound **10b**

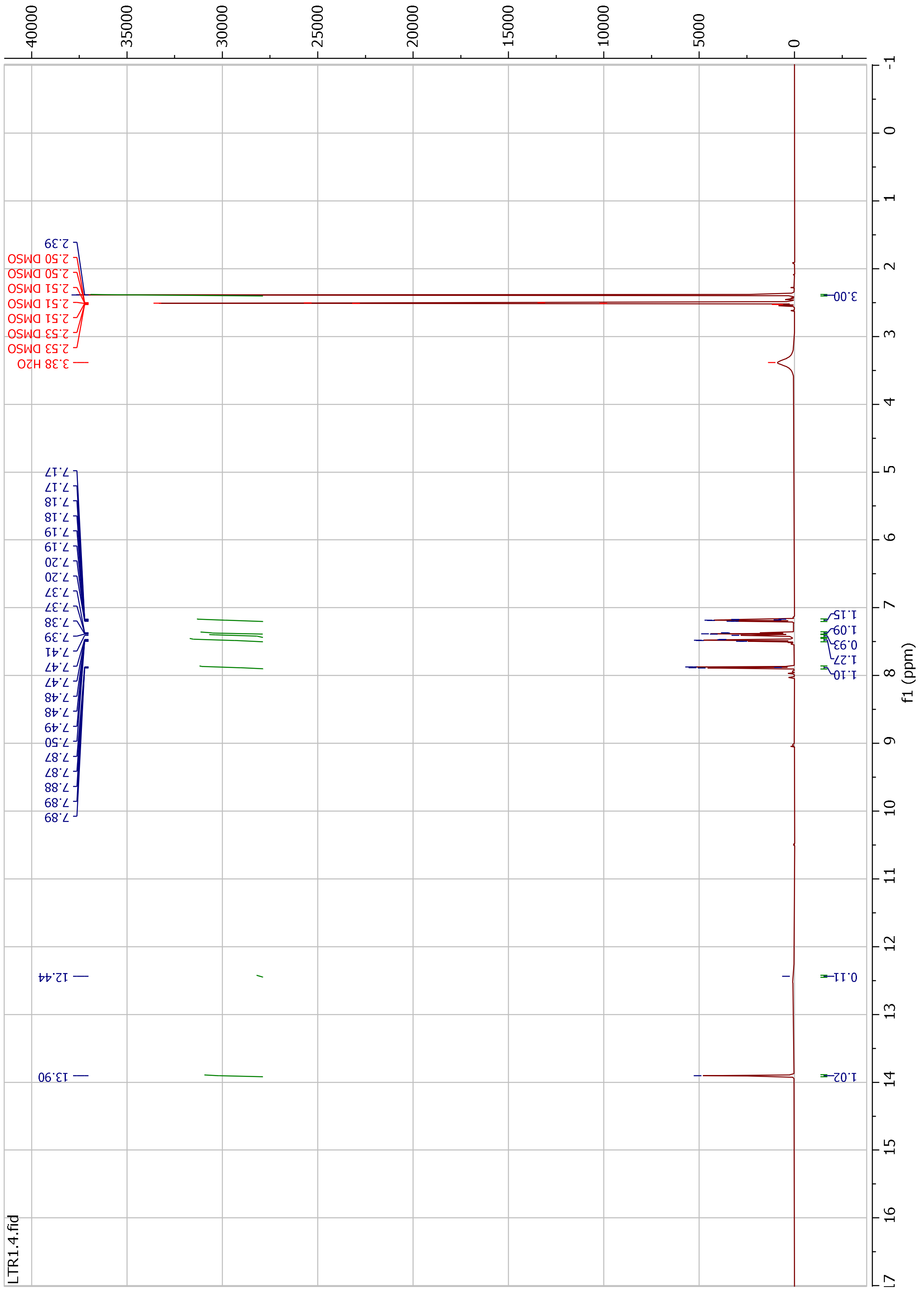

Compound **11b**

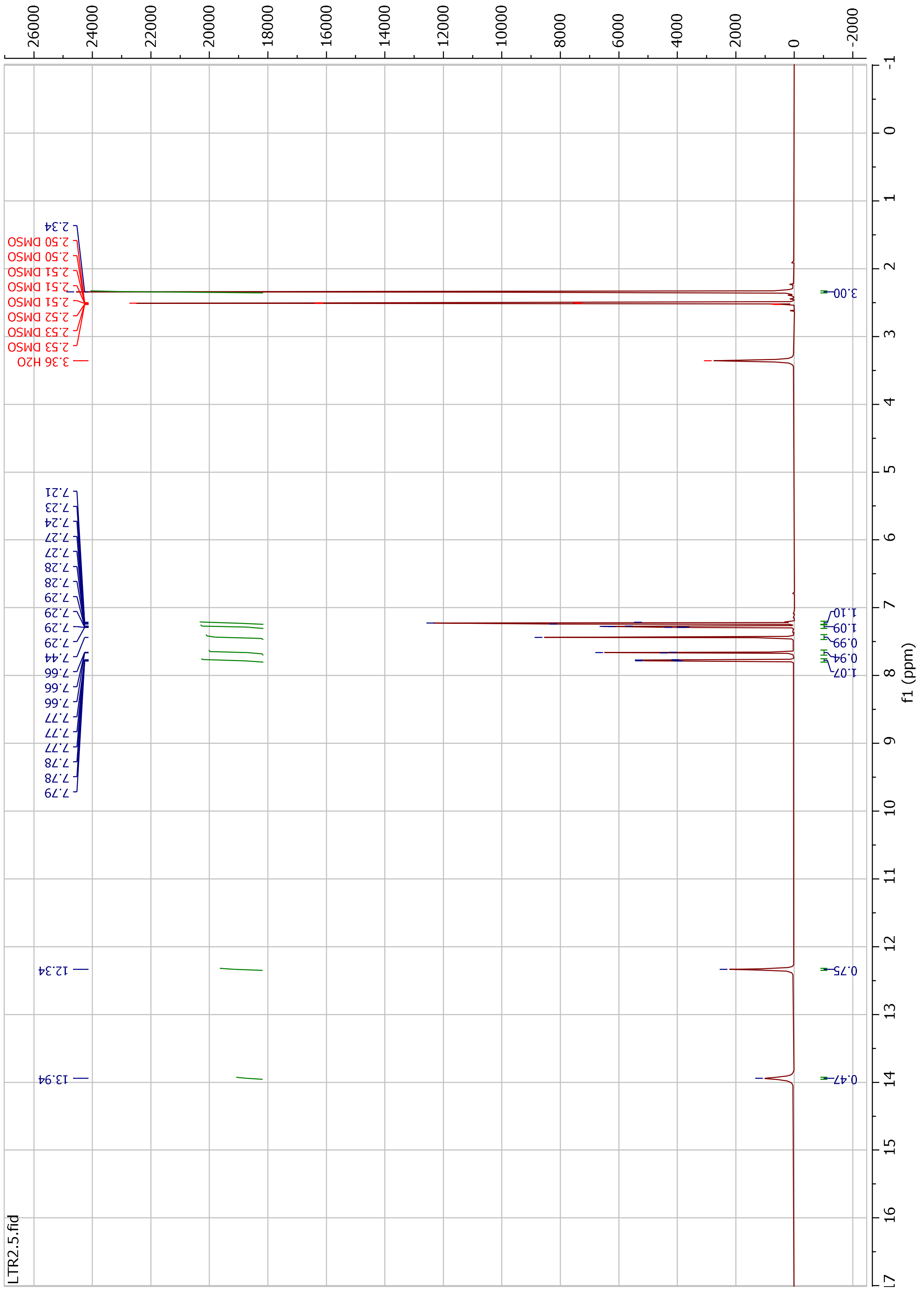

Compound **12b**

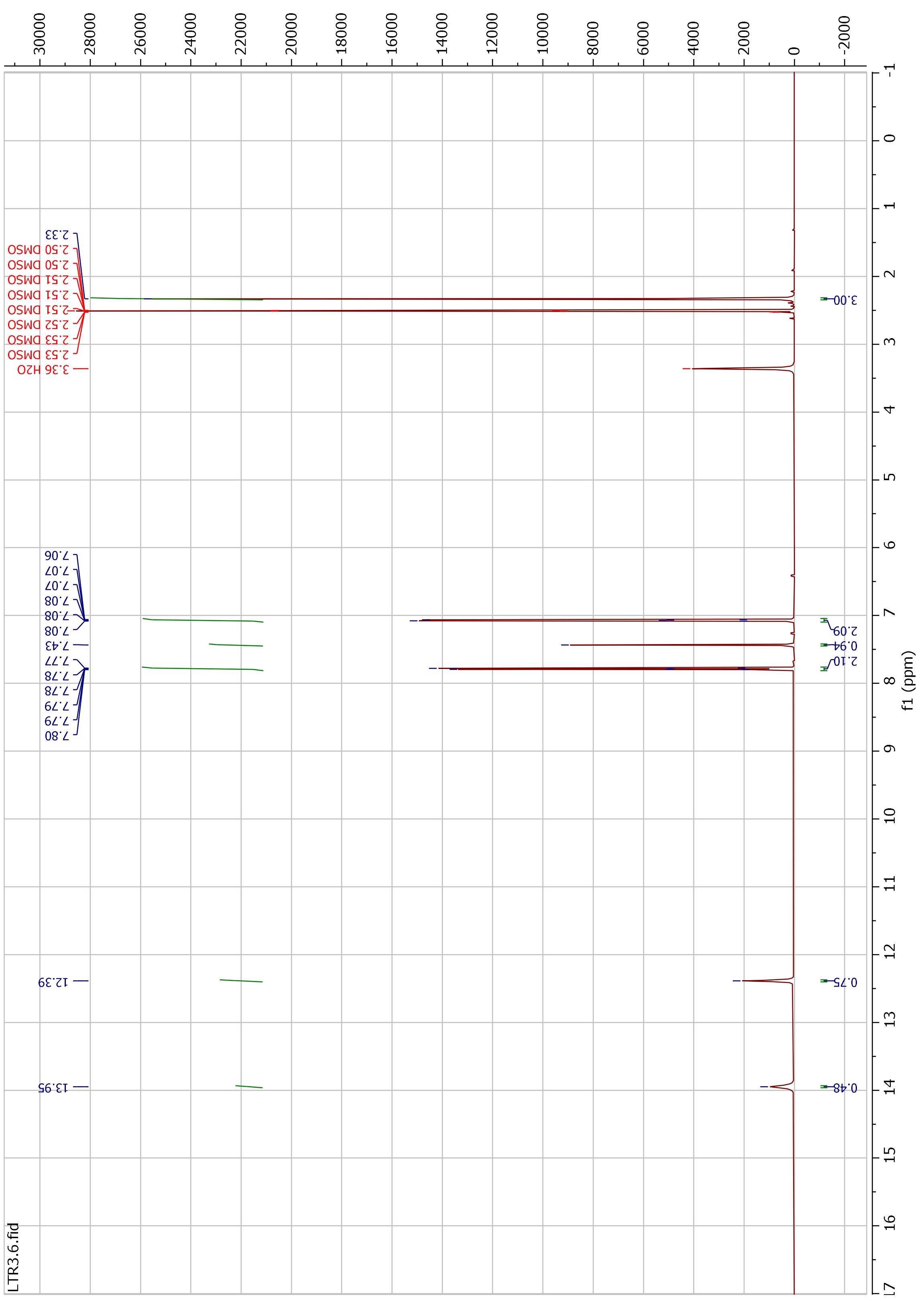

Compound **13b**

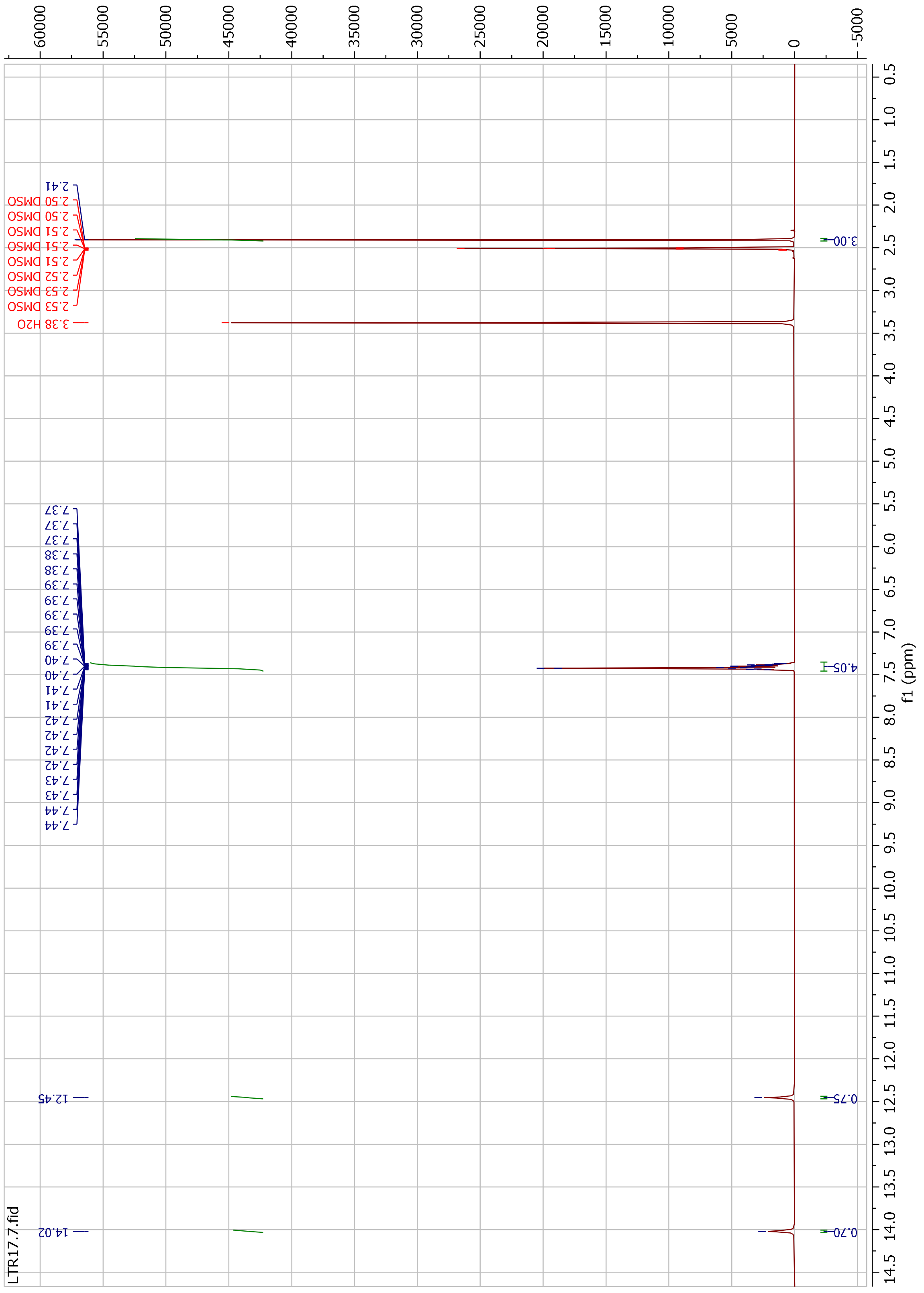

Compound **14b**

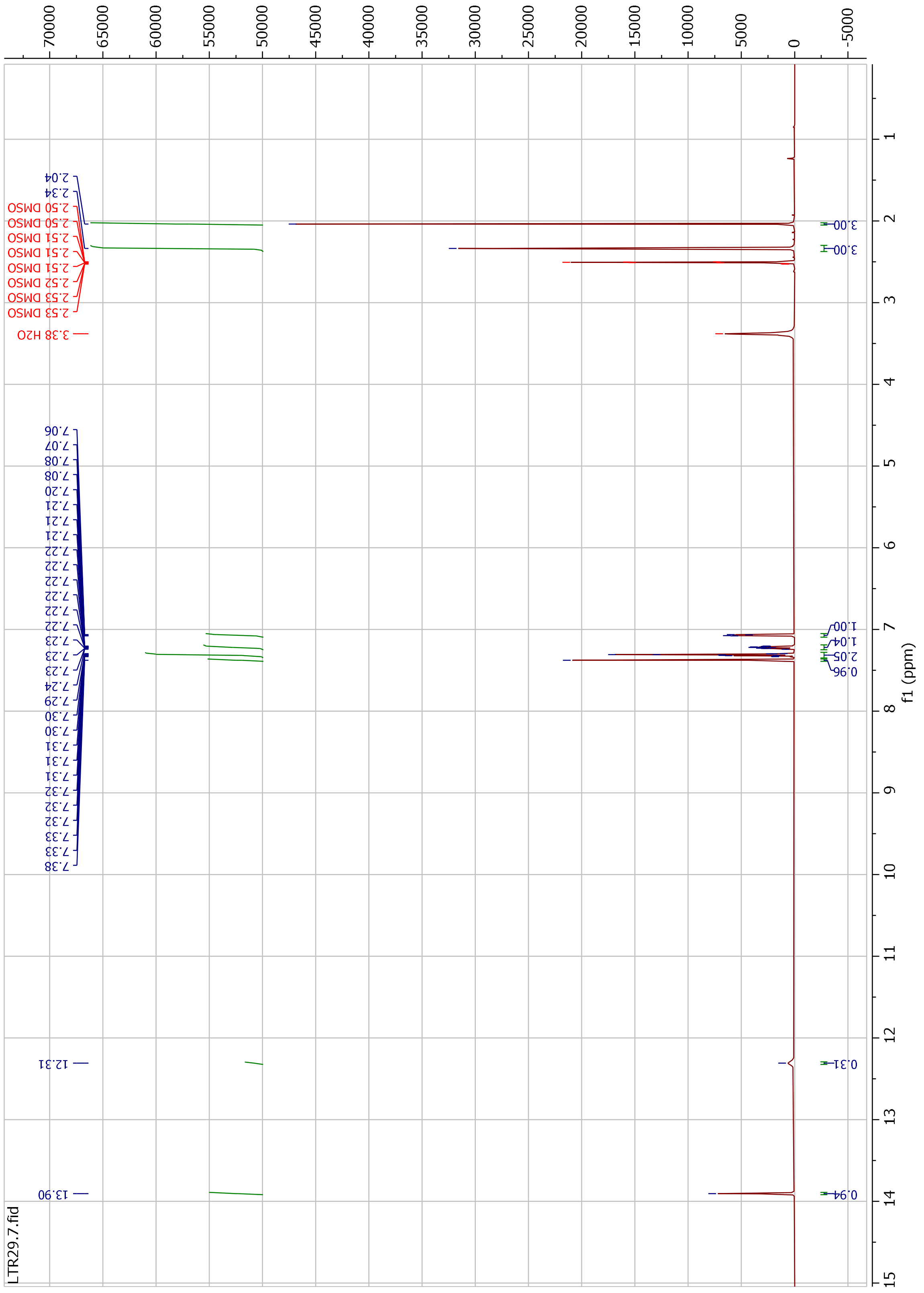

Compound **15b**

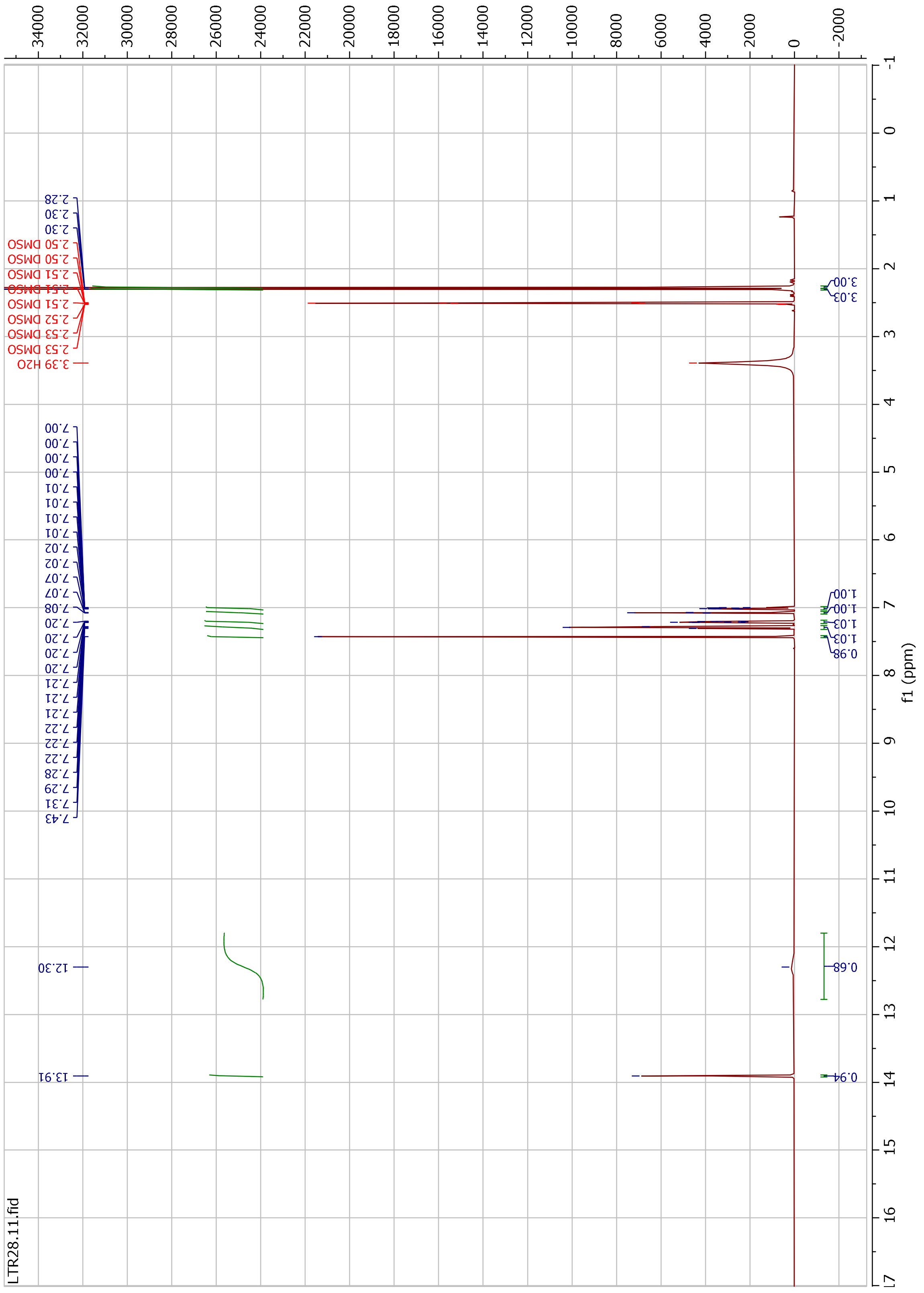

Compound **17b**

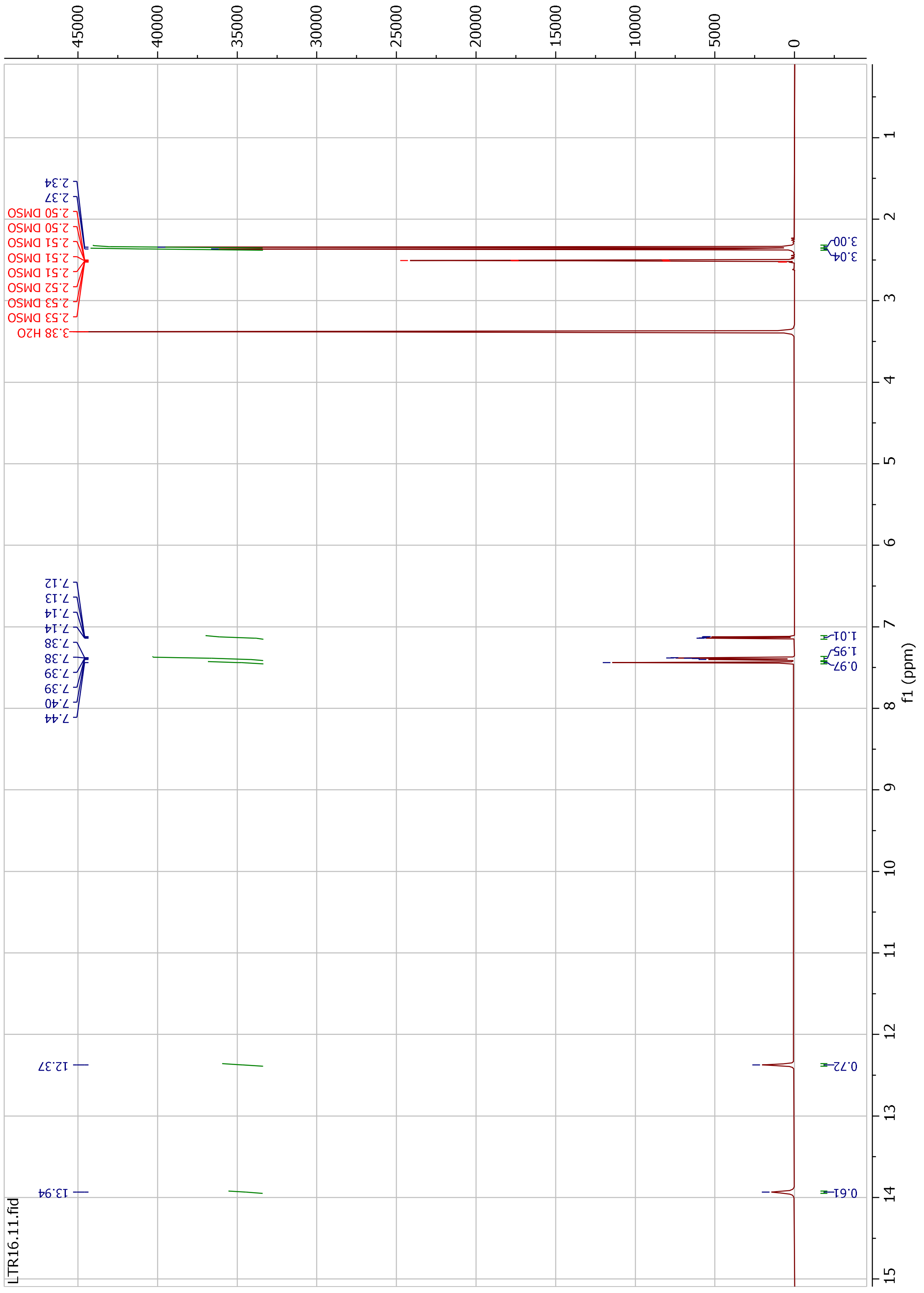

Compound **18b**

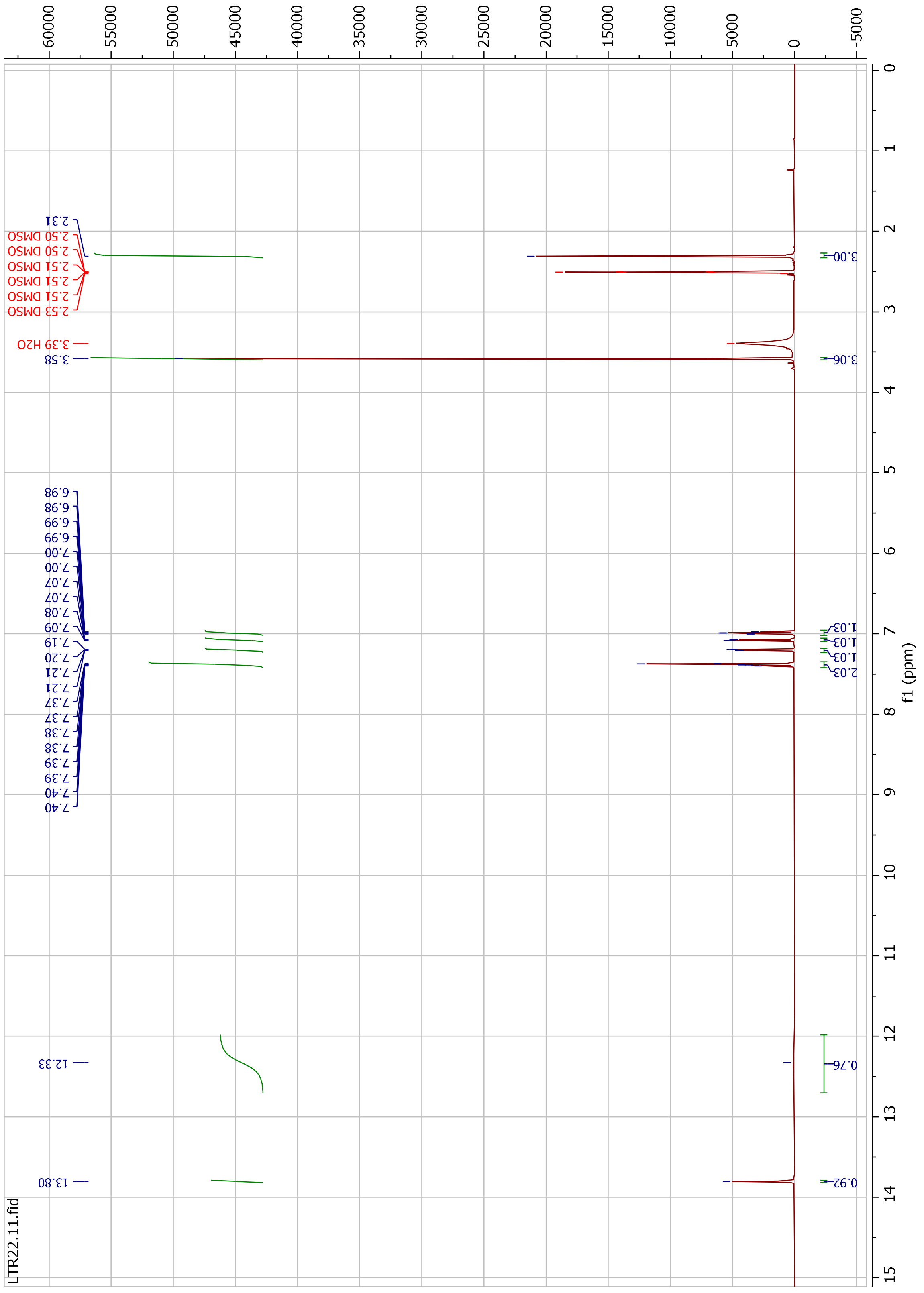

Compound **19b**

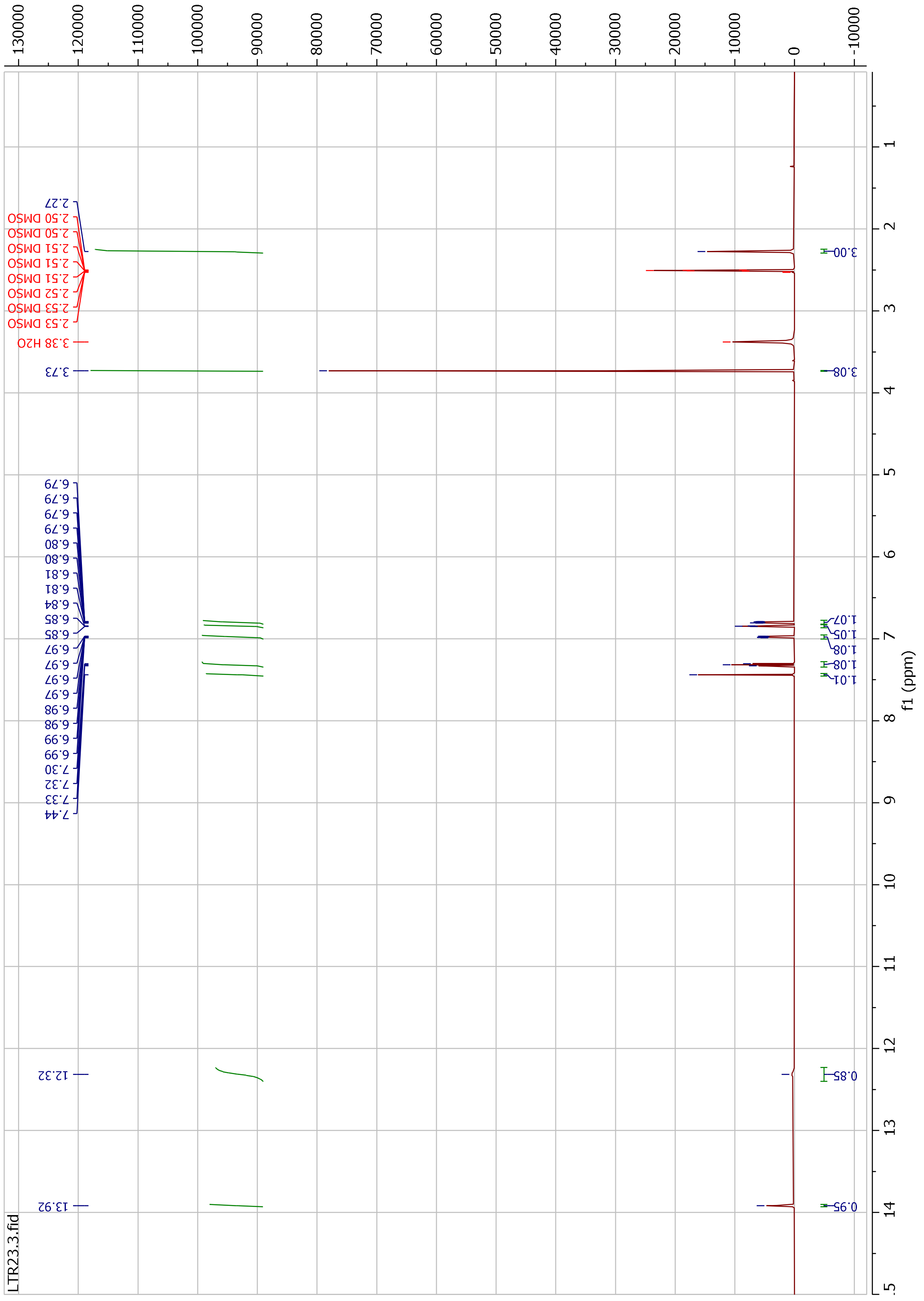

Compound **21b**

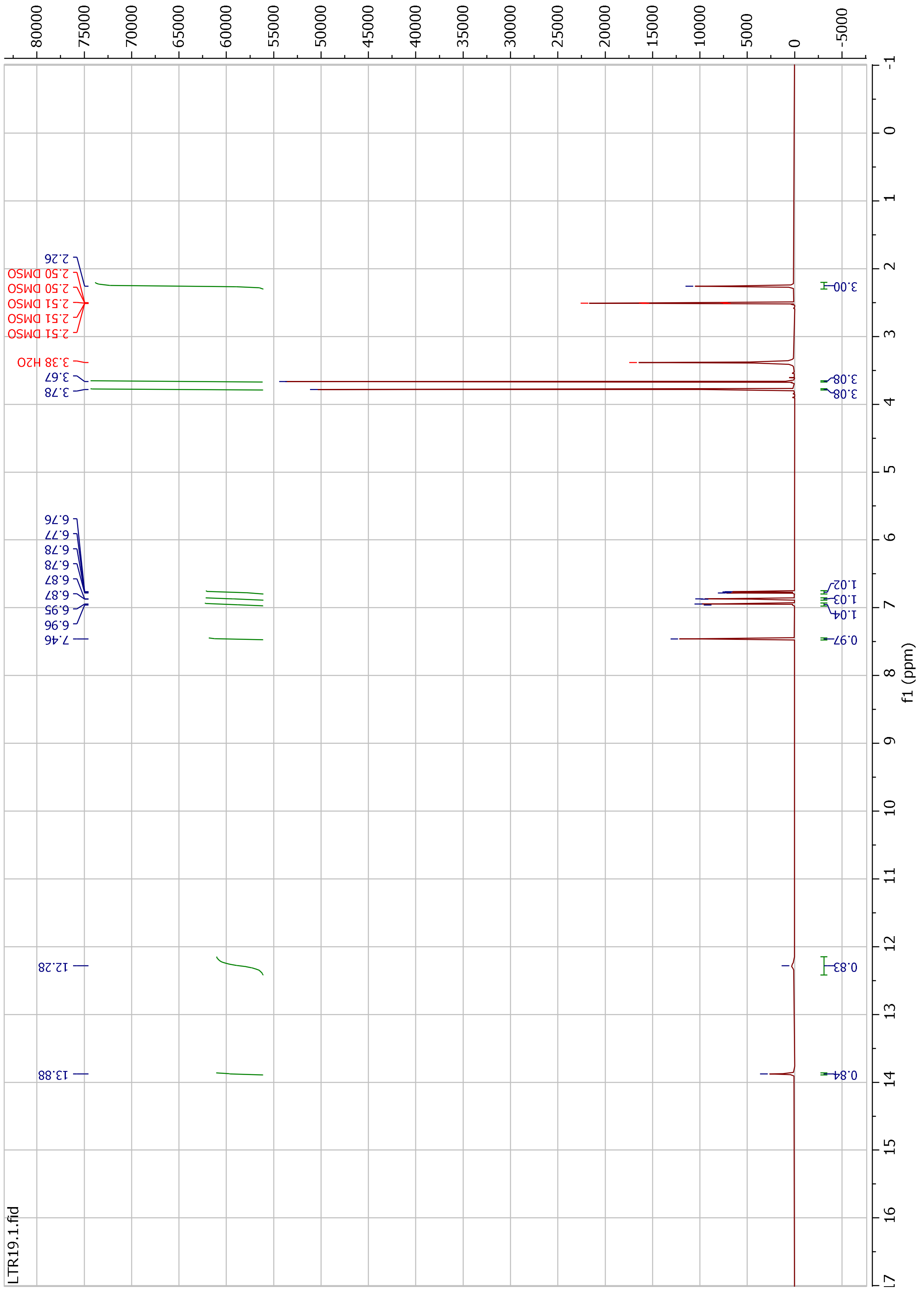

Compound **22b**

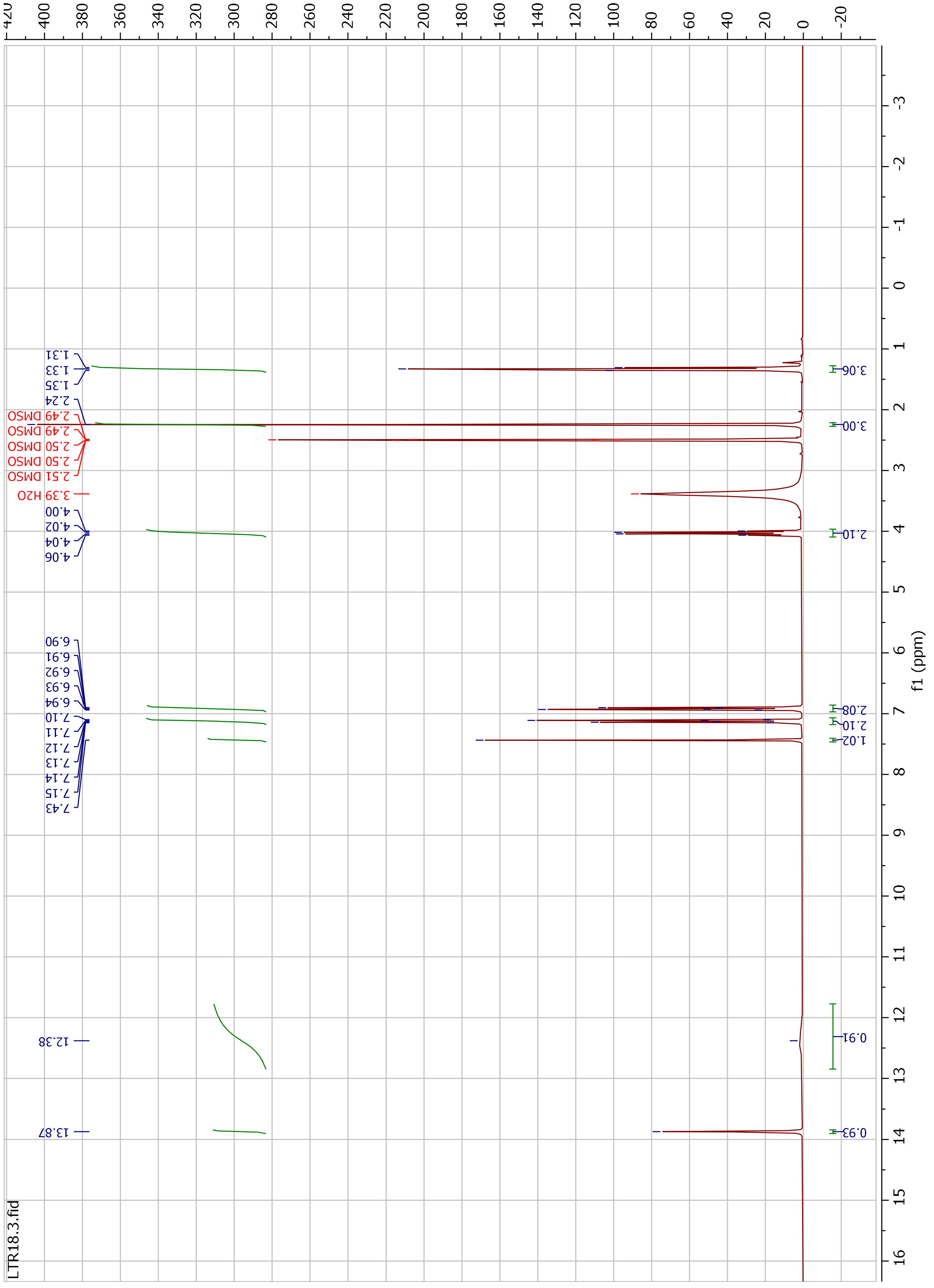

Compound **23b**

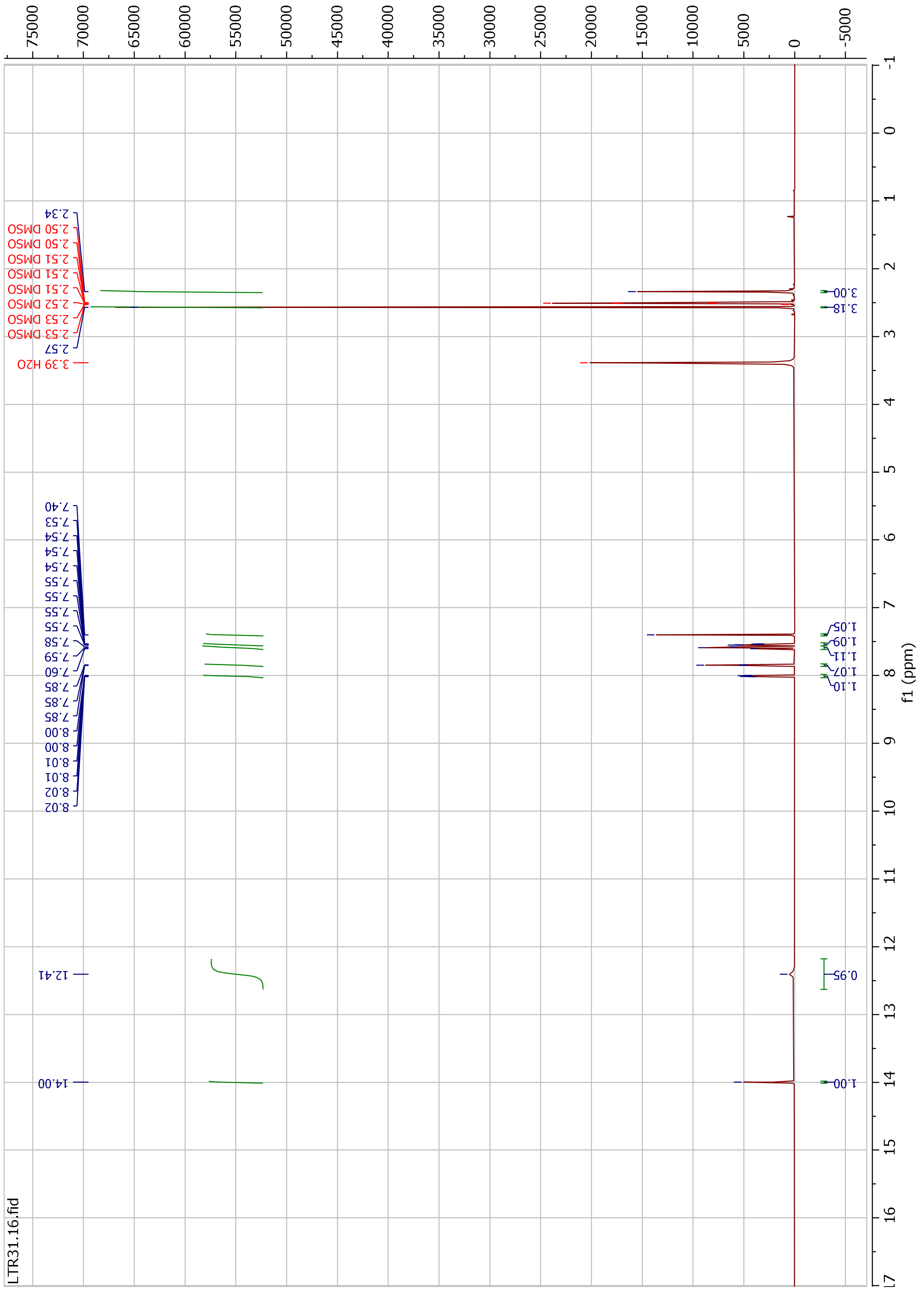

Compound **24b**

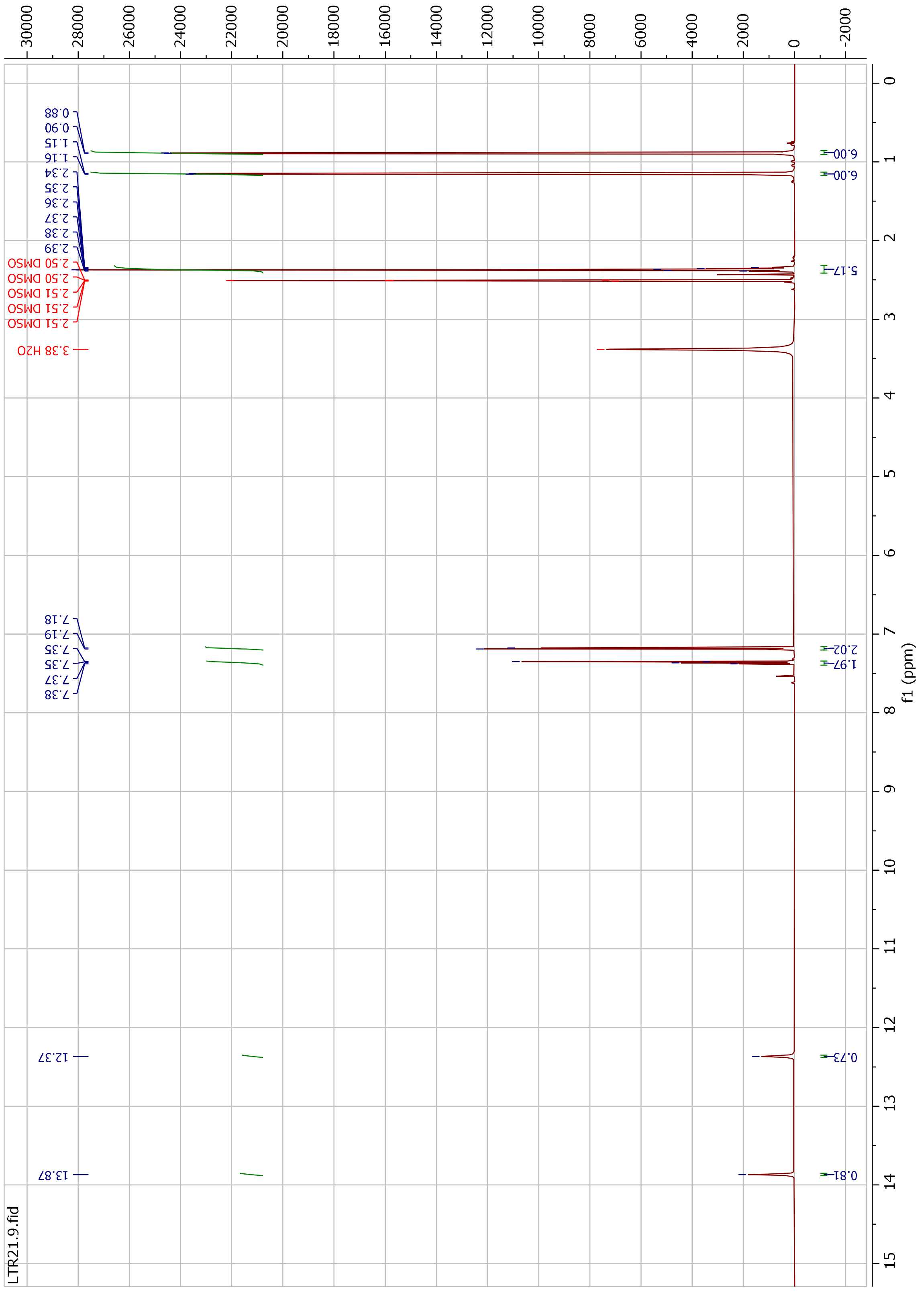

Compound **25b**

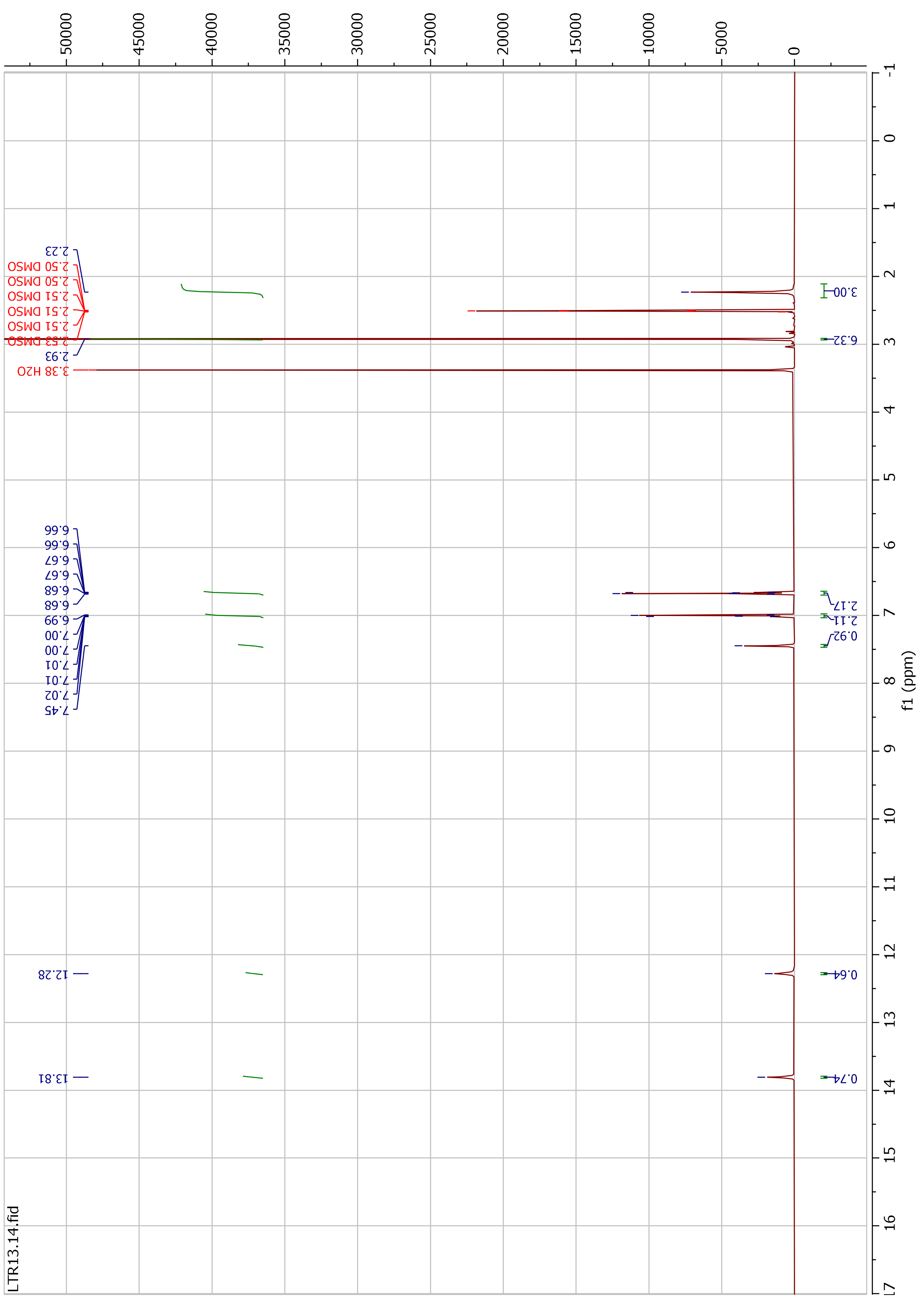

Compound **26b**

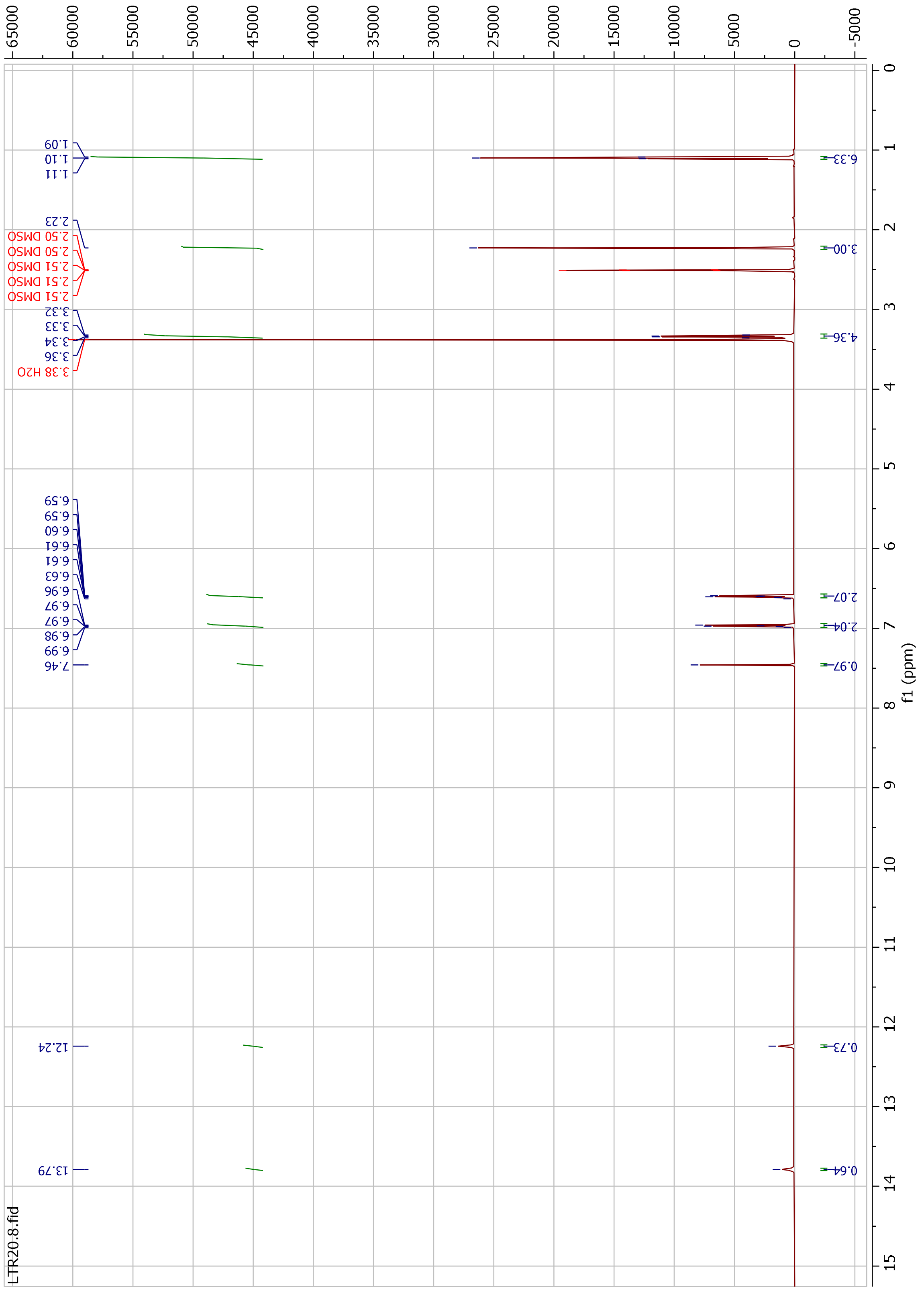

Compound **27b**

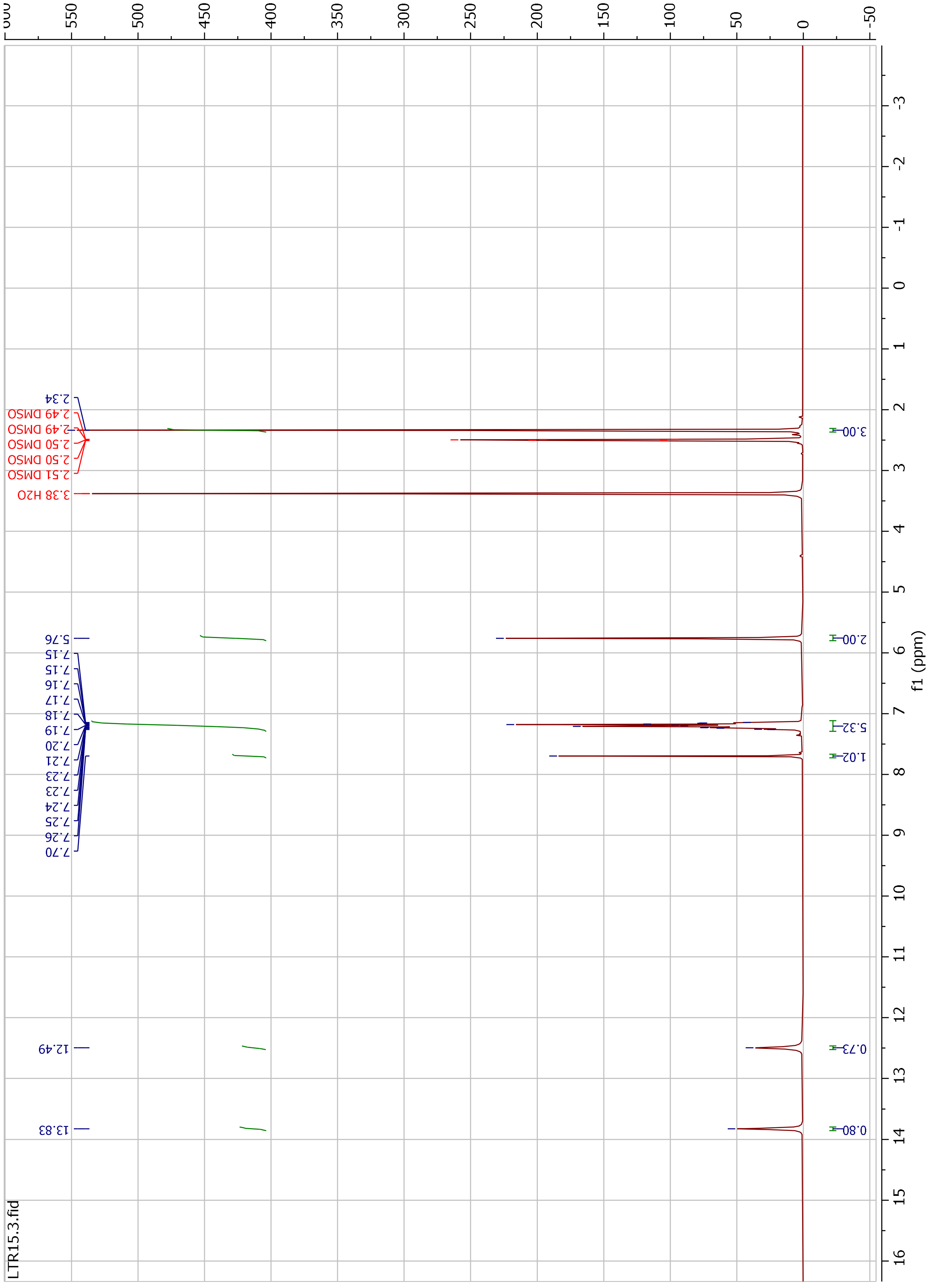

Supplement: Supplemental Material [file IENZ_A_2112576_SM5687.pdf]
